# Androgen receptor‐mediated transcriptional repression targets cell plasticity in prostate cancer

**DOI:** 10.1002/1878-0261.13164

**Published:** 2022-02-02

**Authors:** Éva Erdmann, Pauline Ould Madi Berthélémy, Félicie Cottard, Charlotte Zoe Angel, Edwige Schreyer, Tao Ye, Bastien Morlet, Luc Negroni, Bruno Kieffer, Jocelyn Céraline

**Affiliations:** ^1^ CNRS UMR 7104 INSERM U1258 IGBMC University de Strasbourg Illkirch France; ^2^ University of Freiburg Freiburg im Breisgau Germany; ^3^ Genomic Medicine Research Group BMSRI Ulster University Belfast UK; ^4^ Institut de Cancérologie de Strasbourg Europe (ICANS) Hôpitaux Universitaires de Strasbourg Strasbourg France; ^5^ Faculté de Médecine Fédération de Médecine Translationnelle de Strasbourg FMTS Université de Strasbourg Illkirch France

**Keywords:** androgen receptor, AR‐V7, cell plasticity, EMT, prostate cancer, transcriptional repression

## Abstract

Androgen receptor (AR) signaling remains the key therapeutic target in the management of hormone‐naïve‐advanced prostate cancer (PCa) and castration‐resistant PCa (CRPC). Recently, landmark molecular features have been reported for CRPC, including the expression of constitutively active AR variants that lack the ligand‐binding domain. Besides their role in CRPC, AR variants lead to the expression of genes involved in tumor progression. However, little is known about the specificity of their mode of action compared with that of wild‐type AR (AR‐WT). We performed AR transcriptome analyses in an androgen‐dependent PCa cell line as well as cross‐analyses with publicly available RNA‐seq datasets and established that transcriptional repression capacity that was marked for AR‐WT was pathologically lost by AR variants. Functional enrichment analyses allowed us to associate AR‐WT repressive function to a panel of genes involved in cell adhesion and epithelial‐to‐mesenchymal transition. So, we postulate that a less documented AR‐WT normal function in prostate epithelial cells could be the repression of a panel of genes linked to cell plasticity and that this repressive function could be pathologically abrogated by AR variants in PCa.

## Graphical abstract

Androgen receptor (AR) is essential for prostate development and function, and also for prostate cancer (PCa) cell survival and proliferation. In this study, we show that the transcriptional repressive program of AR in PCa is a determinant of constrained cell plasticity and that this protective role is lost in the presence of pathological constitutively active AR variants or during androgen ablation therapy.

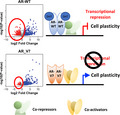

**Abbreviations**
AR: androgen receptor
AREs: androgen‐responsive elements
AR‐WT: wild‐type AR
BioID2: proximity‐dependent biotin identification‐2
BP: biological process
CC: cellular component
CDH2: cadherin‐2 (N‐cadherin) gene
CRPC: castration‐resistant prostate cancer
DHT: dihydrotestosterone
EMT: epithelial‐to‐mesenchymal transition
EtOH: ethanol
FBS: fetal bovine serum
FC: fold change
FDR: false discovery rate
GEO: Gene Expression Omnibus
GO: Gene Ontology
GSEA: gene set enrichment analysis
HRP: horseradish peroxidase
MF: molecular function
MSigDB: Molecular Signature Database
nAREs: negative AREs
PCa: prostate cancer

## 1 Introduction

With an estimated 1.27 million newly diagnosed men worldwide in 2018, prostate cancer (PCa) remains the second most common cancer in men according to the GLOBOCAN project and is the fifth leading cause of death from cancer in men, which places it as a vitally important public health issue [1]. PCa cell growth and survival rely on the bio‐availability of androgens, such as testosterone and its derived dihydrotestosterone (DHT), whose action is mediated by androgen receptor (AR) [2, 3, 4].

Androgen receptor is a ligand‐dependent transcription factor that belongs to the nuclear receptor superfamily [2, 5]. In a schematic view, in the absence of a ligand, AR is localized in the cytoplasm, folded by chaperon proteins in an inactive but ligand‐binding competent state [6]. Following ligand binding, AR translocates to the nucleus and binds to androgen‐responsive elements (AREs) present in enhancer, superenhancer, introns, and/or promoter of target genes [7]. Thereafter, AR recruits pioneer factors and cofactors that favor chromatin opening, and then gene expression [8, 9, 10, 11]. This landscape of AR cistrome can be pathologically reprogrammed in human prostate cancer [12, 13, 14].

Androgen receptor remains a key therapeutic target in the management of hormone‐naïve‐advanced PCa and CRPC [15, 16]. However, the efficacy of androgen deprivation therapy is transient as all patients will ultimately relapse [17, 18]. Several molecular mechanisms can drive to CRPC [19], and most of them maintain in an active state AR signaling pathways [17]. Indeed, nonsense mutations and diverse *AR* gene rearrangements that result in the expression of constitutively active AR variants emerge as important molecular mechanisms that lead to CRPC [20, 21, 22, 23, 24]. Besides their role in therapeutic response, AR variants seem to play a key role in prostate cancer progression to a more aggressive stage. Indeed, AR‐Q641X and AR‐V7, but not AR‐WT, lead to the upregulation of mesenchymal markers, in particular, N‐Cadherin, Vimentin, Snail, and ZEB1 [25]. Moreover, contrary to constitutively active AR variants, AR‐WT binding to AREs present in *CDH2* intron 1 following dihydrotestosterone stimulation is not accompanied by an upregulation of N‐cadherin [26]. Altogether, these data suggest that AR‐WT and constitutively active AR variants control differently the expression of a panel of genes at the transcriptional level.

To go deeper in this hypothesis, we performed in the present study RNA‐seq and proteomic analyses, as well as cross‐analyses of experimental data with other publicly available PCa cell transcriptomic datasets to decipher a full landscape on distinctive transcriptional activities of AR‐WT, AR‐V7, and AR‐Q641X in PCa cells. We found that DHT‐activated AR‐WT inhibited the expression of a panel of genes and that this property was pathologically lost with the expression of constitutively active AR variants. We further showed that the panel of repressed genes by DHT‐activated AR‐WT encoded for effectors of cell membrane and cell adhesion functions. So, we postulate that one of the expected normal functions of AR in prostatic tissue could be to prevent the expression of genes linked to cell plasticity. As cell plasticity is closely linked to tumor progression, it may be interested to pay attention to long‐term consequences of AR targeting on PCa cell feature.

## 2 Materials and methods

### 2.1 Cell culture

Stable transduced LNCaP (clone FGC; ECACC, *European Collection of Authenticated Cell Cultures*) and C4‐2B (ViroMed Laboratories, Burlington, NC, USA) cells were used to obtain doxycycline‐inducible expression of eGFP‐AR‐WT (AR‐WT), eGFP‐AR‐Q641X (AR‐Q641X), eGFP‐AR‐V7 (AR‐V7), or eGFP alone (control) as previously described [26]. Cells were maintained in RMPI‐1640 complete medium supplemented with 10% of tetracycline‐system‐approved fetal bovine serum (FBS) (BD Biosciences, Le Pont de Claix, France), 10 mm HEPES, 2 mm L‐glutamine, 1 mm sodium pyruvate (Invitrogen, Life Technologies SAS, Courtaboeuf, France), 100 U·mL^−1^ penicillin, 100 μg·mL^−1^ streptomycin (Sigma‐Aldrich, Saint‐Quentin‐Fallavier, France), 200 μg·mL^−1^ geneticin, and 400 ng·mL^−1^ puromycin (Life Technologies SAS).

### 2.2 RNA extraction

Transduced LNCaP and C4‐2B cells were seeded in RPMI‐1640 complete medium with charcoal‐treated FBS and without geneticin and puromycin. After 48 h, cells were treated for 24 h with 20 ng·mL^−1^ doxycycline to induce the expression of AR‐WT, AR‐Q641X, AR‐V7, or eGFP alone. Also, as indicated, cells were concomitantly treated with 10 nm DHT or ethanol (EtOH) as vehicle. Total RNA was isolated using NucleoSpin® RNA II assay (Macherey‐Nagel, Hoerdt, France) according to the manufacturer’s protocol. This experiment was performed in three biological replicates for each condition.

### 2.3 RNA sequencing

RNA samples obtained from transduced LNCaP were used for library preparation and high‐throughput sequencing on the Illumina Hiseq 4000 as Single‐Read 50 base reads were performed by the GenomEast genomic platform (IGBMC, Illkirch, France). Reads were mapped onto hg38 assembly of human genome using tophat v2.0.14 [27] and bowtie2 v2.1.0 aligner [28]. Quantification of gene expression was performed using htseq v0.6.1 [29] and gene annotations from Ensembl release 84.

### 2.4 Source and information of publicly available RNA‐seq data

Three GEO RNA‐seq datasets, GSE125014 [30], GSE148397 [31], and GSE151429 [32], were downloaded from the publicly available Gene Expression Omnibus (GEO) database (https://www.ncbi.nlm.nih.gov/geo/). GSE125014 dataset refers to LNCaP cells treated with 10 nm DHT or 10 μm enzalutamide (MDV3100) for 4 and 24 h. GSE148397 dataset concerns VCaP cells treated with 1 nm R1881 alone or together with 500 nm (low) or 2 μm (high) darolutamide for 8 and 22 h. GSE151429 dataset refers to LNCaP cells expressing in a doxycycline manner ARv567es, a constitutively active AR variant. Transcriptomic changes were evaluated 24 h after culturing in a steroid‐free medium containing 30 ng·mL^−1^ doxycycline.

### 2.5 Data normalization and differential gene expression analysis on DESeq2

All raw expression data were normalized and implemented in the DESeq2 Bioconductor library (DESeq2 1.30.0) on r (version 4.0.3) with default settings [33, 34]. Comparisons of interest were performed in order to obtain the (base 2) log of the fold changes (log2FC) and the corresponding adjusted *P*‐values. Indeed, from our experimental dataset, differentially expressed genes were calculated either between DHT‐treated control or AR‐WT expressing cells and vehicle‐treated control, or between vehicle‐treated AR‐Q641X or AR‐V7 expressing cells and vehicle‐treated control as indicated.

From GSE125014 dataset, differentially expressed genes were calculated between DHT‐ or enzalutamide‐ and vehicle‐treated LNCaP cells. From GSE148397 dataset, differentially expressed genes were calculated between R1881‐ or R1881 plus darolutamide‐ and vehicle‐treated VCaP cells. From GSE151429 dataset, differential gene expression was calculated between doxycycline‐treated cells and vehicle‐treated cells in the absence of androgen. To take into account the large dispersion observed with low read counts and to obtain more accurate log2FC estimates, shrinkage of the estimates (*lfcShrink* function) was applied using ‘apeglm’ (version 1.12.0) as type of shrinkage estimator [35].

For all RNA‐seq data, log2FC results are expressed as the mean ratio of the indicated number of observations for each condition. *P*‐values were adjusted for multiple testing using the guideline of Benjamini and Hochberg [36], and differences were considered statistically significant when *P*‐value < 0.05. Differentially expressed genes were defined according to the following criteria: *P*‐value < 0.05 and Log2FC > 1 or < −1 for the present study, GSE125014 and GSE151429 datasets, and *P*‐value < 0.01 and Log2FC > 2 or < −2 for the GSE148397 dataset.

### 2.6 Functional enrichment analysis

To identify significantly enriched pathways in different experimental conditions, gene set enrichment analysis (GSEA) was performed on preranked datasets sorted by log2FC using the gsea 4.1.0 desktop application [37, 38]. A conservative scoring approach was defined by setting the scoring scheme parameter to *classic* (unweighted). Gene set used for analysis with GSEA was the Molecular Signature Database (MSigDB) hallmark collection (v7.2). The GSEA Preranked tool provides for each gene set of the collection an enrichment score that reflects how often members of that gene set occur at the top or bottom of the ranked dataset. Then, the score was normalized for each gene set to account for the size of the set. Only results with a false discovery rate (FDR) *q*‐value < 0.25 were considered significant, as defined by the publishers of the GSEA tool, and presented ranked by their GSEA normalized enrichment score. To further analyze pathways and biological functions that could be specifically associated with AR‐repressive activity, significantly downregulated genes in the three datasets were pooled. The resulting panel of AR‐repressed genes was uploaded on the Gene Ontology (GO) Consortium’s Web site (http://geneontology.org/) for overrepresentation analysis among the three GO categories *biological process* (BP), *molecular function* (MF), and *cellular component* (CC) [39, 40]. Results with a *P*‐value < 0.05 and a fold enrichment > 2 were selected as significant, and the first ten enriched terms of each category were presented according to their *P*‐value. Furthermore, the MSigDB hallmark collection was used on the Enrichr online tool (https://maayanlab.cloud/Enrichr/) for the enrichment analysis of the repressed genes. This approach allows the identification of significant overlaps between our list of genes and a gene set of the collection [41, 42]. Significant enriched gene sets (*P*‐value < 0.05) were presented ranked by *P*‐value with indication of the number of overlaps.

### 2.7 Reverse transcription and real‐time PCR

Reverse transcription was conducted from 500 ng of total RNA using iScript Reverse Transcription Supermix for RT‐qPCR kit (Bio‐Rad, Marnes‐la‐Coquette, France) as recommended by the manufacturer. Then, real‐time PCR was performed with GoTaq® qPCR Master Mix (Promega, Charbonnières‐les‐Bains, France) and validated primers for *TMPRSS2*, *KLK3*, *ITGA3*, *HDAC9*, *COL16A1*, *SMARCD3,* and *ITGB4* (Quantitect primer assay, Qiagen, Courtaboeuf, France; Table S1). The housekeeping gene *HMBS* (QT00014462, Qiagen) expression was used to normalize the results according to the $2^{\Delta C_{t}}$ method.

### 2.8 Time‐course proteomic analysis

Stably eGFP‐AR‐WT expressing LNCaP cells were seeded in complete medium and then starved during 48 h in RPMI‐1640 complete medium with charcoal‐treated FBS before treatment with 10 nm DHT or vehicle. After 24 and 48 h, cells were collected by centrifugation and washed 4 times in phosphate‐buffered saline. Sample preparation and mass spectrometry were performed at the IGBMC proteomic platform. Briefly, cell pellets containing about 2 × 10^6^ cells were lysed in 1% SDS, 0.1 m Tris pH 8.5, 50 mm DTT, and sonicated.

### 2.9 Screen for androgen receptor partner interactions

In order to analyze a potential difference in partner recruitment between AR‐WT and constitutively active AR variants, we used the previously described proximity‐dependent biotin identification (BioID2) approach [43]. Plasmids pMyc‐BioID2‐AR‐WT, pMyc‐BioID2‐AR‐Q641X, and pMyc‐BioID2‐AR‐V7, in which AR‐WT or AR variants were fused to the N‐ter of the humanized Aquifex aeolicus BioID2 protein, were constructed from pMyc‐BioID2 (#74223, Addgene, Teddington, UK). Then, Myc‐BioID2‐AR‐WT, Myc‐BioID2‐AR‐Q641X, or Myc‐BioID2‐AR‐V7 fragments were inserted in pLVX‐TRE3G from the Tet‐On 3G‐inducible expression lentiviral system (Takara Bio Europe, Saint‐Germain‐en‐Laye, France) to yield a doxycycline‐inducible expression of the respective transgenes in LNCaP cells. The unfused Myc‐BioID2 transgene was considered as control. For biotinylating AR partners, 1.5 × 10^6^ transduced cells were plated in p100 dishes for 48 h up to 80% of cell confluence, and then, cells were placed in fresh medium containing 2 ng·mL^−1^ doxycycline, 50 μm of biotin, and 10 nm DHT. After 24 h, cells were lysed in the RIPA lysing and extraction buffer supplemented with 25 U·mL^−1^ de benzonase and 1X Protease Inhibitor Cocktail. Cell extracts were then sonicated (50% Amplitude, 10 s Pulse‐ON, 20 s Pulse‐OFF; Q700 ultrasonic processor, QSonic, Newtown, CT, USA) and clarified by at 14 000 ***g*** at 4 °C for 15 min. Biotinylated proteins were purified using the streptavidin‐affinity approach. Briefly, lysates were incubated overnight at 4 °C and under agitation in 200 μL magnetic streptavidin‐coupled beads (Invitrogen™ Dynabeads™, Thermo Fisher Scientific, Waltham, MA, USA). After centrifugation, beads were washed twice in 2% SDS, once in RIPA, twice in a washing buffer containing 10% glycerol, 50 mm HEPES‐NaOH pH 8, 150 mm NaCl, 2 mm EDTA, and 0.1% NP‐40, and then resuspended in 85 μL Laemmli buffer. Biotinylated proteins were then eluted from beads at 98 °C for 5 min and separated in a 7.5% SDS–PAGE. All experiments were repeated three times on separate days.

### 2.10 Mass spectrometry analysis

Protein samples were reduced, alkylated, and digested at 37 °C for AR interactome analysis, or double digested with Lys‐C and trypsin at 37 °C for time‐course proteomic approach. Peptide mixtures were then desalted on C18 spin‐column and dried on Speed‐Vacuum before LC‐MS/MS analysis. Peptides were analyzed using an Ultimate 3000 nano‐RSLC (Thermo Scientific, San Jose, CA, USA) coupled in line with an LTQ‐Orbitrap ELITE mass spectrometer via a nano‐electrospray ionization source (Thermo Scientific). Briefly, peptides were loaded in triplicate on a C18 Acclaim PepMap100 trap‐column (Thermo Fisher Scientific) and then separated on a C18 Accucore nanocolumn (Thermo Fisher Scientific) with linear gradients of acetonitrile and analyzed in TOP20 CID data‐dependent MS method. For the time‐course proteomic approach, proteins were identified by database searching using Sequest‐HT (Thermo Fisher Scientific) with Proteome Discoverer 2.4 software (PD2.4, Thermo Fisher Scientific) on Homo Sapiens database (SwissProt, reviewed, release 2020_04_06, 20286 entries). Oxidation and carbamidomethylation were set as variable and fixed modification, respectively. Peptides were filtered with an FDR at 1%, rank 1, and proteins were identified with a minimum of 2 unique peptides. The Label‐Free Quantification was based on the XIC (Extracted Ion Chromatogram), where protein abundancies were calculated from the average of peptide abundancies using the TOP N (where *N* = 3, the 3 most intense peptides for each protein), and only the unique peptide was used for the quantification. Quantification values were exported in Perseus for statistical analysis [44]. For AR interactome analysis, proteins were identified by database searching against human database using Maxquant 1.6.5.0. Precursor and fragment mass tolerance before recalibration were set at 20 ppm and 0.6 Da, respectively. Trypsin was set as enzyme, and up to two missed cleavages were allowed. Carbamidomethylation was set as fixed modification, oxidation, and N‐term acetylation as variable modifications. Proteins were identified with a minimum of two unique peptides and were filtered with an FDR < 1. Normalization and quantitative values (iBAQ) were processed with Perseus 1.6.2.0.

### 2.11 BioID interactome analysis

After the normalization step of mass spectrometry data, differences in AR partners were then calculated between AR‐Q641X and AR‐WT or AR‐V7 and AR‐WT conditions. To identify partners that have been specifically lost in the presence of AR variants, differentially underrepresented proteins (*P*‐value < 0.05 and Log2FC < −1) were searched from intersected AR‐Q641X or AR‐V7 and AR‐WT data. The list of interactors underrepresented in the presence of AR variants compared with AR‐WT was then submitted to Gene Ontology analysis as described above (2.6). Identification of experimentally proved AR interactors was performed using the BioGRID database (https://thebiogrid.org) [45].

### 2.12 Western Blot analysis

Cells were lysed in RIPA buffer (Pierce, Fisher Scientific, Illkirch, France) supplemented with 1× protease inhibitor cocktail (Roche, Meylan, France) and 25 U·mL^−1^ Benzonase (Millipore, Molsheim, France) during 20 min on ice. Protein concentration was quantified using Bio‐Rad Protein Assay (Bio‐Rad) and according to the manufacturer’s protocol. Western blot analysis was performed as previously described with a starting amount of 40 μg of protein for each condition [26]. Membranes were probed with primary antibodies against AR (1 : 1000, catalog no. 554225, BD Biosciences), β‐tubulin III (1 : 4800, catalog no. T2200, Sigma‐Aldrich), c‐myc clone 9E10 (1 : 1000, catalog no. 13‐2500, Thermo Fisher Scientific) as indicated at 4 °C overnight, then with horseradish peroxidase‐conjugated rat anti‐mouse (1 : 1000, catalog no. 553391, BD Biosciences) or goat anti‐rabbit (1 : 2000, catalog no. 7074, Cell Signaling Technology) secondary antibodies at room temperature for 1 h. For biotinylated proteins, membranes were probed after blocking with streptavidin‐horseradish peroxidase (HRP) (1 : 40 000, catalog no. 21130, Pierce) at room temperature for 1 h.

### 2.13 Statistics

qPCR results represent mean ± standard error of the mean (SEM) of three biological repeats. Statistical analysis was performed using Student’s *t*‐test by comparing the control, AR‐WT, AR‐Q641X, or AR‐V7 condition versus the eGFP condition treated with vehicle, and *P*‐values < 0.05 (*) were considered to be statistically significant.

### 2.14 Data analysis and graphic representation

All data analysis and visualization were performed in python 3 [46] using the pandas, bokeh, matplotlib, numpy, scipy, and seaborn packages.

## 3 Results

### 3.1 Distinctive transcriptomic program of constitutively active AR variants in prostate cancer cells

We have previously reported dual transcriptional activities between constitutively active AR and wild‐type AR (AR‐WT) in prostate cancer [26]. Constitutively active AR variants have been involved in the expression of mesenchymal markers, while on the contrary, AR‐WT seems to impede such expression. Our previous data suggest that AR‐WT may play an occluding function to prevent the expression of mesenchymal markers as evidenced for *CDH2* expression. To delineate molecular mechanisms involved in this duality, we first compared the global transcriptome profile triggered by AR‐WT with those of constitutively active AR variants, AR‐V7 and AR‐Q641X. RNA‐seq was then performed in the androgen‐sensitive LNCaP cells, which express an endogenous AR containing the T878A mutation. LNCaP cells were then lentivirally transduced to co‐express in a doxycycline‐dependent manner either AR‐WT, AR‐Q641X, or AR‐V7 in fusion with eGFP. LNCaP cells expressing eGFP alone and treated with vehicle were considered as control to calculate the log_2_ fold change in gene expression between different experimental conditions (Fig. S1). We first validated our model by analyzing DHT‐induced gene expression modifications in control, and in LNCaP cells expressing AR‐WT. Twenty‐four hours after DHT treatment, 183 genes were downregulated and 466 were overexpressed in control, indicating transcriptional activities of the T878A endogenous AR in LNCaP cells (Table 1).

**Table 1:** Significant differentially expressed genes. LNCaP cells were transduced to express AR‐WT, AR‐Q641X, AR‐V7, or the empty eGFP plasmid (control) and were exposed to DHT or vehicle (EtOH) as indicated. Gene expression levels in the presence of DHT‐activated AR‐WT, AR‐Q641X_EtOH, or AR‐V7_EtOH (Datasets A) were compared with the reference dataset B corresponding to Control_EtOH. The number of differentially expressed genes with adjusted p‐value < 0.05 and |log_2_FC| > 1 is indicated. The number of under‐ and overexpressed genes, A < B and A > B, respectively, is also indicated.

Condition	RNA‐seq datasets comparison (A vs B)	Number of differentially expressed genes	Number of underexpressed genes (A < B)	Number of overexpressed genes (A > B)
Control (eGFP)	Control_DHT vs Control_EtOH	649	183	466
AR‐WT	AR‐WT_DHT vs Control_EtOH	860	395	465
AR‐Q641X	AR‐Q641X_EtOH vs Control_EtOH	1558	78	1480
AR‐V7	AR‐V7_EtOH vs Control_EtOH	1091	71	1020

**Fig. 1:**
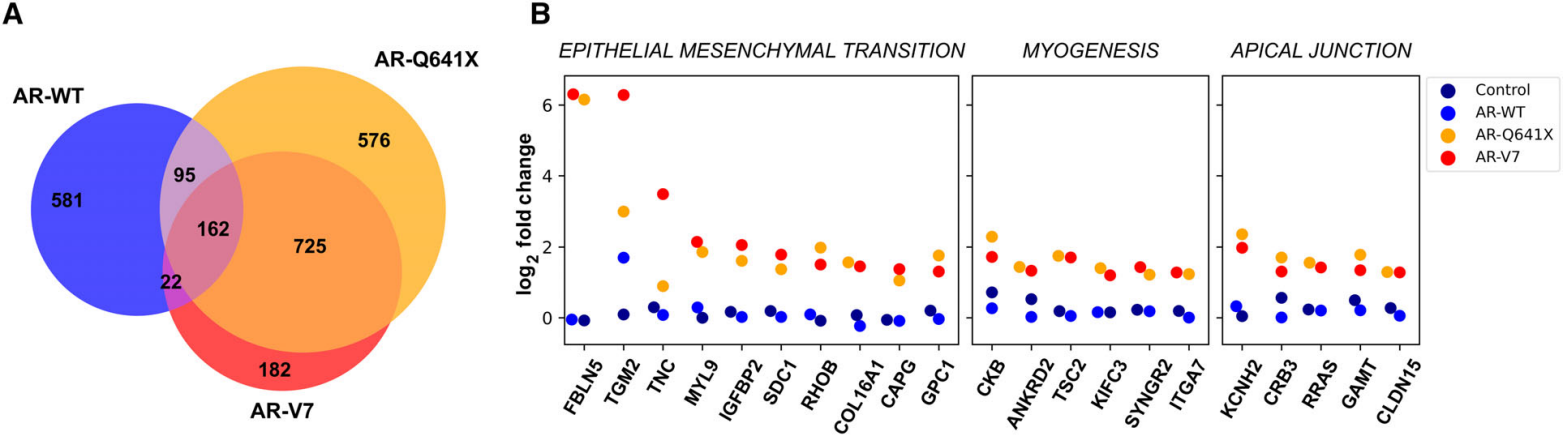
Differential transcriptome of AR‐WT and constitutive AR variants, AR‐Q641X and AR‐V7, in LNCaP cells. (A) Venn diagram comparing the sets of AR‐WT, AR‐Q641X, and AR‐V7 significantly differentially expressed genes (*n* = 3). Genes with *P*‐value adjusted for multiple testing < 0.05 and |log2FC| > 1. (B) Differential expression of genes involved in ‘epithelial–mesenchymal transition’, ‘myogenesis’, and ‘apical junction’.

A panel of 30 known androgen‐responsive genes in prostate tissue epithelium was used to further validate the experimental model [47] (Fig. S1). When focused on DHT‐activated AR‐WT, the number of down‐ and upderegulated genes markedly shifted to 395 and 465, respectively. Besides, about 1558 and 1091 genes were retrieved deregulated (|log2FC| > 1; *P*‐value < 0.05) in the presence of AR‐Q641X or AR‐V7, respectively (Fig. 1A; Table 1). Noteworthily, only 17% of these genes (257 out of 1558 and 184 out of 1091, respectively, for AR‐Q641X and AR‐V7) were common with those deregulated in the presence of DHT‐activated AR‐WT (Fig. 1A).

Altogether, these data indicate that AR‐Q641X and AR‐V7 do not completely mirror those of DHT‐liganded AR‐WT or AR‐T878A in LNCaP cells, and highlight again differential transcriptional activities between constitutively active AR variants and DHT‐activated full‐length AR. It was also interesting to note that when assessed at the same time, in the same cellular model, AR‐V7 shared with AR‐Q641X only 57% (887 out of 1558; |log2FC| > 1; *P*‐value < 0.05) of deregulated genes, suggesting further transcriptional specificity among these two AR variants (Fig. 1A).

### 3.2 AR variants exhibited reduced transcriptional repression activities in prostate cancer cells

**Fig. 2:**
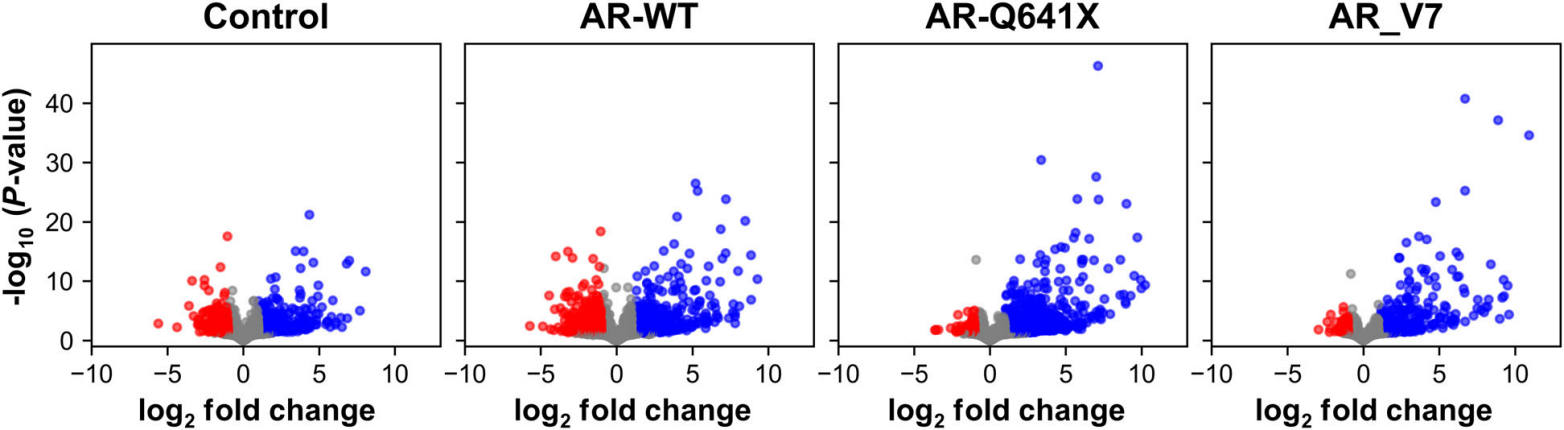
Distribution of RNA‐seq data. Volcano plot representing the distribution of RNA‐seq data of the four experimental conditions. Genes with adjusted *P*‐value < 0.05 and |log_2_FC| > 1 are shown in red (significantly downregulated genes) and blue (significantly upregulated genes). RNA‐seq was performed from three biological replicates.

To further investigate differential gene regulation between full‐length AR and constitutively active AR variants, we focused on their transcriptional repression activities. Transcriptional repression activity that was evident in control and AR‐WT co‐expressing cells appeared to be lost in the presence of AR‐Q641X and AR‐V7 (Fig. 2; Table 1). Indeed, nearly 28% and 46% of deregulated genes were downregulated, respectively, in control and in the presence of AR‐WT. Besides, the percentage of repressed genes dropped to 5% and 6% in the presence constitutively active AR‐Q641X and AR‐V7, respectively (Fig. 2; Table 1). We next wondered whether, ARv567es, another constitutively active AR variant elicited similar results. We then used a publicly available RNA‐seq GEO dataset, GSE125014, obtained in LNCaP cells expressing ARv456es in a doxycycline‐inducible manner. A similar asymmetry of down‐ and upregulated genes was also observed (Fig. S2), reinforcing our idea that compared with AR‐WT, transcriptional repression capacities of constitutively active AR variants are disturbed.

**Fig. 3:**
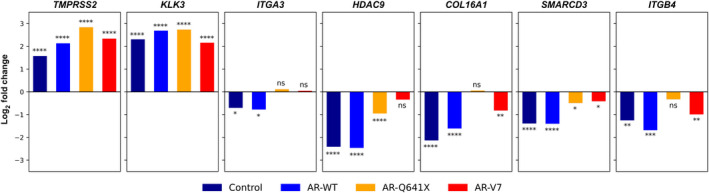
Loss of transcriptional repression activities of AR variants AR‐Q641X and AR‐V7 in LNCaP cells. Gene expression was analyzed by qPCR. The log2Fold change in gene expression was calculated between the four experimental conditions and the control (eGFP) cells treated with vehicle as reference. Bar graphs represent mean of 3 biological repeats. Student’s *t*‐test was used to compare control, AR‐WT, AR‐Q641X, or AR‐V7 condition with the eGFP condition treated with vehicle. *P < 0.05, **P < 0.01, ***P < 0.001, ****P < 0.0001, ns, nonsignificant.

In this light, the following gene panel, *TMPRRSS2*, *KLK3*, *ITGA3*, *HDAC9*, *COL16A1*, *SMARCD3,* and *ITGB4* was selected to further validate by RT‐qPCR this dual transcriptional regulation between DHT‐activated AR‐WT and constitutively active AR variants in LNCaP and in C4‐2B cells. As expected, *TMPRSS2* and *KLK3* were positively regulated in all conditions, and the drop in the expression level of *ITGA3*, *HDAC9*, *COL16A1*, *SMARCD3,* and *ITGB4* observed with DHT‐activated AR‐WT was significantly attenuated in cells expressing constitutively active AR variants (Fig. 3; Fig. S3).

We next checked by realizing a time‐course proteomic analysis in LNCaP cells whether the repressive transcriptional activity of DHT‐activated AR‐WT was noticeable at the protein level. Indeed, a significant and time‐dependent increase in the number of underrepresented proteins (|log_2_FC| > 0.6; *P*‐value < 0.05) was observed, including a panel of 14 proteins for which the corresponding encoding gene was part of the downregulated genes in the presence of DHT‐activated AR‐WT (Fig. S4, Table S2). Together, these data suggest that following DHT activation, AR‐WT could trigger the downregulation of a specific panel of genes and that this property would be pathologically lost by constitutively active AR variants.

### 3.3 Similar AR‐binding sites associated with both up‐ and downtranscriptional regulation by DHT‐activated wild‐type AR

A possible molecular mechanism to explain differences in transcriptional repressive activities between AR‐WT and constitutively active AR variants could be distinctive recognition of so‐called negative AREs (nAREs) [48]. However, to go deeper in this hypothesis, we first wondered whether there was a difference in AR‐binding sites associated with transcriptional activation and repression by DHT‐activated AR. So, available AR ChIP‐seq data from LNCaP and VCaP cells were downloaded from GEO database and intersected with the lists of up‐ and downregulated genes. Sequences corresponding to AR‐binding sites (500 bp centered on the peak summit) were subsequently retrieved from human reference genome (hg19/GRCh37) and submitted to MEME‐ChIP webserver (https://meme‐suite.org/meme/tools/meme‐chip) for motif analysis (Fig. S5). FOXA1 and AR motifs were the first DNA sequence motifs that were similarly found in AR‐binding sites associated with up‐ and downregulated genes by DHT‐activated AR in LNCaP and VCaP cells (Fig. S6). These data suggest that a difference in DNA sequence of AR‐binding sites was not linked to gene downregulation by AR‐WT, and prompted us to investigate rather differences in regulatory complexes formed around DHT‐activated AR‐WT and constitutively active AR variants.

### 3.4 AR variants disengaged from corepressor recruitment

**Fig. 4:**
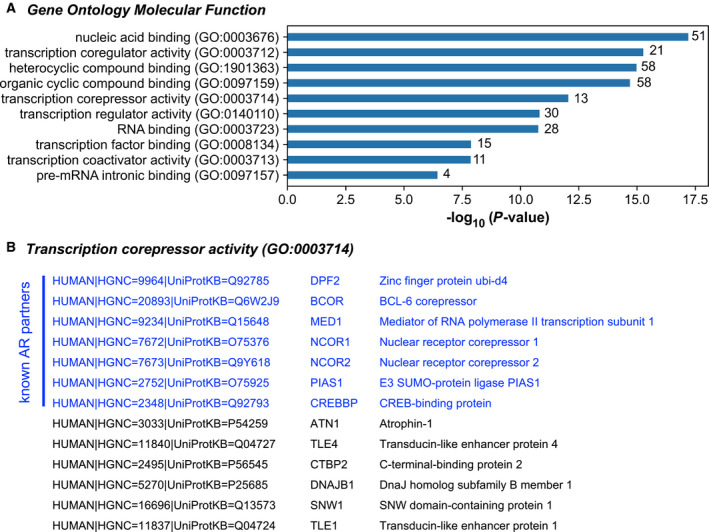
BioID analysis of AR interactome. (A) Gene ontology (GO) analysis of 78 potential partners identified by mass spectrometry using the BioID2 approach and underrepresented in AR variants conditions compared with AR‐WT. (B) Among the 13 proteins related to the ‘transcription corepressor activity’ GO term, 7 are known as experimentally proved AR partners (BIOGRID database).

As AR ligand‐binding domain and AF‐2 are known to present interfaces for recruitment of numerous coregulators, the lack of the C‐terminal part in constitutively active AR variants could lead to the formation of particular complexes, explaining therefore the decrease in their transcriptional repression capacities. To analyze regulatory complexes formed around AR‐WT and constitutively active AR variants, the BioID2 approach was applied (Fig. S7). A mass spectrometry analysis was thereafter carried out from purified biotinylated proteins from myc‐BioID2, myc‐BioID2‐AR‐WT, myc‐BioID2‐AR‐Q641X, and myc‐BioID2‐AR‐V7 transduced LNCaP cells. After raw data normalization, 78 biotinylated proteins were underrepresented in the presence of myc‐BioID2‐AR‐Q641X and myc‐BioID2‐AR‐V7 compared with myc‐BioID2‐AR‐WT. An enrichment analysis indicated that these 78 potential AR partners fit mainly in GO molecular function terms around transcriptional regulation, including ‘*nucleic acid binding*’, ‘*transcription coregulator activity*’, ‘*heterocyclic compound binding*’, ‘*organic cyclic compound binding*’, ‘*transcription corepressor activity*’, and ‘*transcription regulator activity*’ (Fig. 4A). According to the BIOGRID database, among the 13 proteins related to ‘*transcription corepressor activity*’, 7 are known as experimentally proved AR partners, including BCOR, NCOR1, NCOR2, and PIAS1 (Fig. 4B).

These data suggest that constitutively active AR variants may lose transcriptional repression capacities due to the building of singular transcriptional regulatory complexes and that this may be linked to the lack of the ligand‐binding domain and AF‐2.

We next inquired about the reason that constitutively active AR variants that are linked to castration‐resistant prostate cancer, the most aggressive stage of the disease, lose their repressive capacities. So, to get insight into key issues involved in AR transcriptional repressive activities in advanced prostate cancer, we decided to further focus on biological and/or molecular signatures associated with our lists of deregulated genes.

### 3.5 The repressive transcriptomic program of wild‐type AR targets cell adhesion features in prostate cancer cells

We first used GSEA tool to analyze hallmarks associated with deregulated genes for each experimental condition. The 10 most upregulated hallmark gene sets in the presence of constitutively active AR‐Q641X and AR‐V7 referred to ‘*myogenesis*’, ‘*androgen response*’, ‘*apical junction*’, and interestingly to ‘*epithelial–mesenchymal transition*’ function (Table 2). Similar hallmark gene sets were associated with transcriptional activities of ARv567es (Fig. S2). These hallmark gene sets associated with cell membrane and migration functions were comforted by the high level of expression of *FBLN5*, *TGM2*, *COL16A1*, *Tuberin (TSC2)*, *Integrin Alpha‐7*, and *RRAS (Ras‐Related)* in the presence of constitutively active AR variants (Fig. 1B). The above‐mentioned hallmark gene sets associated with constitutively active AR variants were not gained in control cells, nor in the presence of DHT‐activated AR‐WT. For these two latter conditions, the following hallmarks ‘*E2F targets*’, ‘*androgen response*’, ‘*MTORC1 signaling*’, ‘*G2M checkpoint*’, ‘*MYC targets*’, and ‘*unfolded protein response*’ were revealed as the ten most significantly upregulated ones (Table 2). This was consistent with the role of DHT‐activated AR in PCa cell proliferation after a period of hormone depletion and in the regulation of unfolded protein response pathways [49, 50].

**Table 2:** Gene Set Enrichment Analysis top positive scores. Ten first upregulated Hallmark gene sets among RNA‐seq data for each condition according to their normalized enrichment score (NES). All enrichment scores have a nominal *P*‐value = 0 and a false discovery rate (FDR) *q*‐value < 0.0005.

Control		AR‐WT		AR‐Q641X		AR‐V7	
HALLMARK	NES	HALLMARK	NES	HALLMARK	NES	HALLMARK	NES
E2F_TARGETS	10.13	E2F_TARGETS	8.15	MYOGENESIS	3.92	MYOGENESIS	3.34
G2M_CHECKPOINT	7.80	ANDROGEN_RESPONSE	7.90	ANDROGEN_RESPONSE	3.42	APICAL_JUNCTION	2.96
ANDROGEN_RESPONSE	6.27	MTORC1_SIGNALING	6.30	APICAL_JUNCTION	3.35	ANDROGEN_RESPONSE	2.72
MYC_TARGETS_V1	4.96	G2M_CHECKPOINT	6.16	HYPOXIA	2.64	MYC_TARGETS_V2	2.64
MYC_TARGETS_V2	4.92	MYC_TARGETS_V1	5.84	ESTROGEN_RESPONSE_EARLY	2.61	UV_RESPONSE_UP	2.49
MTORC1_SIGNALING	4.47	MYC_TARGETS_V2	5.09	EPITHELIAL_MESENCHYMAL_TRANSITION	2.47	GLYCOLYSIS	2.47
UNFOLDED_PROTEIN_RESPONSE	3.32	UNFOLDED_PROTEIN_RESPONSE	4.65	TNFA_SIGNALING_VIA_NFKB	2.43	ESTROGEN_RESPONSE_EARLY	2.35
MITOTIC_SPINDLE	2.58	GLYCOLYSIS	3.69	GLYCOLYSIS	2.40	EPITHELIAL_MESENCHYMAL_TRANSITION	2.31
DNA_REPAIR	2.31	UV_RESPONSE_UP	3.07	ESTROGEN_RESPONSE_LATE	2.05	HYPOXIA	2.28
CHOLESTEROL_HOMEOSTASIS	2.06	OXIDATIVE_PHOSPHORYLATION	3.02	UV_RESPONSE_UP	1.97	TNFA_SIGNALING_VIA_NFKB	2.27

**Fig. 5:**
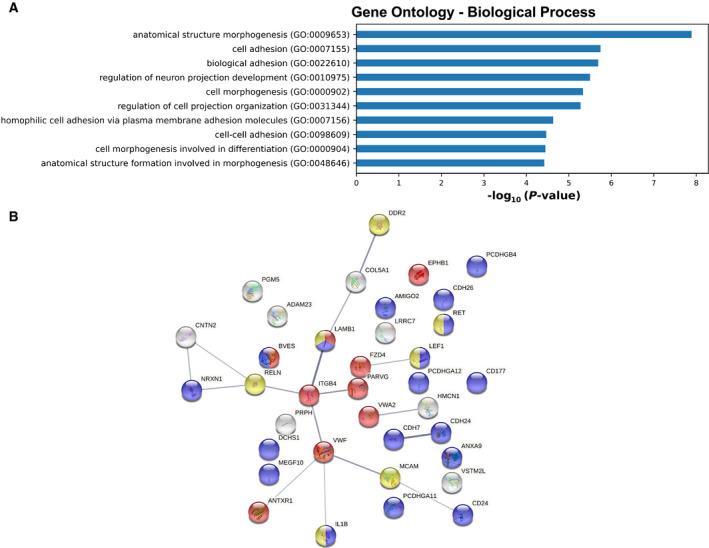
Gene Ontology enrichment analysis of 395 AR‐repressed genes. (A) Bar charts showing the Gene Ontology (GO) terms with a fold enrichment > 2 and *P*‐value < 0.05, for Biological Process (BP) following analysis of the 395 genes repressed by AR‐WT. (B) StringDB network analysis of the 36 genes identified in the ‘cell adhesion’ GO term. The edges indicate both functional and physical protein associations. Colors indicate the proteins related to the ‘cell–cell adhesion’ (blue), ‘cell‐substrate adhesion’ (red), and ‘positive regulation of cell migration’ (yellow) GO BP term.

We next considered cellular and molecular functions of the panel of downregulated genes in the presence of DHT‐activated wild‐type AR. A Gene Ontology enrichment analysis revealed ‘*anatomical structure morphogenesis’*, ‘*cell adhesion*’, ‘*biological adhesion*’, ‘*regulation of neuron projection development*’, and ‘*cell morphogenesis*’ as the top five biological processes associated with the 395 downregulated genes in the presence of DHT‐activated AR‐WT (Fig. 5; Table S3). These results lead us to postulate that following DHT activation, AR‐WT could ultimately trigger to the repression of a panel of genes and that some of these genes would be involved in cell plasticity.

**Fig. 6:**
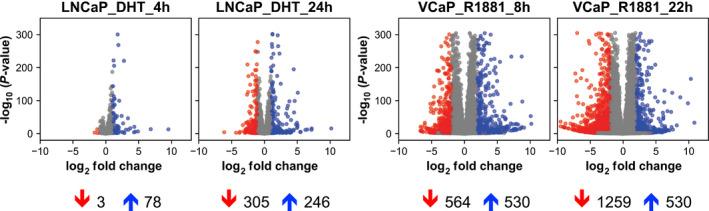
Analysis of repression activity by AR in GEO datasets GSE 125014 and GSE 148397. Volcano plot representing the distribution of GEO datasets with downregulated genes shown in red and upregulated genes shown in blue. (*Left*) GSE125014, thresholds *P*‐value < 0.05 and |log_2_FC| > 1. (*Right*) GSE148397, thresholds *P*‐value < 0.01 and |log_2_FC| > 2. Number of significantly regulated genes are indicated below the plots.

To strengthen our hypothesis, we further investigated the profile of transcription repression by full‐length AR in available RNA‐seq datasets from prostate cancer cells. We choose GSE125014 and GSE148397 datasets originating from LNCaP cells expressing the T878A mutant AR and from VCaP cells expressing a wild‐type AR, respectively. LNCaP cells were stimulated with 10 nm DHT for 4 and 24 h, and VCaP cells were stimulated with 1 nm R1881 for 8 or 22 h. In order to compare our dataset to those of GSE125014 and GSE148397, all data files have been processed with DESeq2 for normalization and identification of differentially expressed genes. As expected, AR‐T878A in LNCaP cells and AR‐WT in VCaP cells triggered to gene repression following ligand stimulation in a time‐dependent manner. The number of downregulated genes in LNCaP cells (*P*‐value < 0.05 and |log_2_FC| > 1) was about 3 and 300 after 4 and 24 h of DHT treatment, respectively. In VCaP cells, significantly downregulated genes (*P*‐value < 0.01 and |log_2_FC| > 2) were about 560 and 1260 after 8 and 22 h of R1881 stimulation, respectively (Fig. 6).

We next used gsea tool to analyze hallmarks associated with deregulated genes for each experimental condition. A time‐dependent change was observed for the top six downregulated hallmark gene sets, particularly for ‘*epithelial–mesenchymal transition*’ (Table 3).

**Table 3:** Analysis of repression activity by AR in GEO datasets. Downregulated Hallmark gene sets that are significantly enriched (FDR *q*‐value < 0.25) among GEO datasets GSE125014 (LNCaP cells) and GSE148397 (VCaP cells) for each indicated condition. Normalized enrichment score (NES) is indicated.

LNCaP_DHT_4h		LNCaP_DHT_24h		VCaP_R1881_8h		VCaP_R1881_22h	
HALLMARK	NES	HALLMARK	NES	HALLMARK	NES	HALLMARK	NES
ESTROGEN_RESPONSE_EARLY	−2.57	KRAS_SIGNALING_DN	−2.92	MYC_TARGETS_V2	−3.87	EPITHELIAL_MESENCHYMAL_TRANSITION	−4.45
MYC_TARGETS_V2	−2.45	HEME_METABOLISM	−2.55	KRAS_SIGNALING_UP	−2.65	KRAS_SIGNALING_DN	−2.51
MYOGENESIS	−2.10	HYPOXIA	−2.41	EPITHELIAL_MESENCHYMAL_TRANSITION	−2.54	KRAS_SIGNALING_UP	−1.89
APICAL_JUNCTION	−1.81	EPITHELIAL_MESENCHYMAL_TRANSITION	−2.18	INFLAMMATORY_RESPONSE	−2.37	COAGULATION	−1.81
INFLAMMATORY_RESPONSE	−1.70	KRAS_SIGNALING_UP	−2.16	INTERFERON_ALPHA_RESPONSE	−1.91	UV_RESPONSE_DN	−1.68
MYC_TARGETS_V1	−1.58	UV_RESPONSE_DN	−2.06	KRAS_SIGNALING_DN	−1.85	ALLOGRAFT_REJECTION	−1.38

**Fig. 7:**
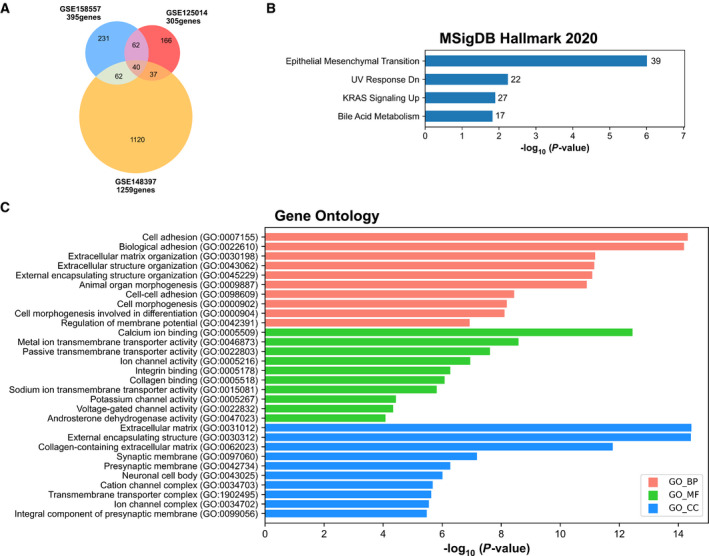
MSigDB Hallmark 2020 and Gene Ontology enrichment analysis of 1718 AR‐repressed genes. (A) Venn diagram representing the intersection of the genes significantly repressed in GSE158557, GSE125014, and GSE148397 data. Genes with adjusted *P*‐value < 0.05 and log2FC < −1 for GSE158557 and GSE125014 data, and adjusted *P*‐value < 0.01 and log2FC < −2 for GSE148397 data. (B) Bar charts showing all significant terms for MSigDB Hallmark 2020 ranked by *P*‐value following analysis of the 1718 repressed gene set. The number of the genes included in the identified pathways is plotted on the right of each bar. (C) Bar charts showing the top 10 Gene Ontology (GO) terms with a fold enrichment > 2 and *P*‐value < 0.05, for biological process (BP), molecular function (MF), and cellular component (CC), following analysis of the 1718 repressed gene set.

From these three RNA‐seq datasets, a total number of 1718 repressed genes were identified (adjusted *P*‐value < 0.05 and log_2_FC < −1 for GSE158557 and GSE125014 data; adjusted *P*‐value < 0.01 and log_2_FC < −2 for GSE148397 data) with partial overlapping (Fig. 7A).

Hallmark gene sets associated with these 1718 repressed genes using the Molecular Signatures Database (MSigDB) and Enrichr software revealed the following hallmarks, ‘*epithelial–mesenchymal transition*’, ‘*UV response Down*’, and ‘*KRAS signaling Up*’ as significant MSigDB terms (Fig. 7B). Gene ontology enrichment analysis further revealed ‘*cell adhesion*’, ‘*biological adhesion, extracellular matrix organization*’ as significant biological processes, ‘*calcium ion binding’*, ‘*integrin binding*’, ‘*collagen binding*’ as significant molecular functions, and finally, ‘*extracellular matrix*’, ‘*collagen‐containing extracellular matrix*’, and ‘*presynaptic membrane*’ as significant cellular components (Fig. 7C).

**Fig. 8:**
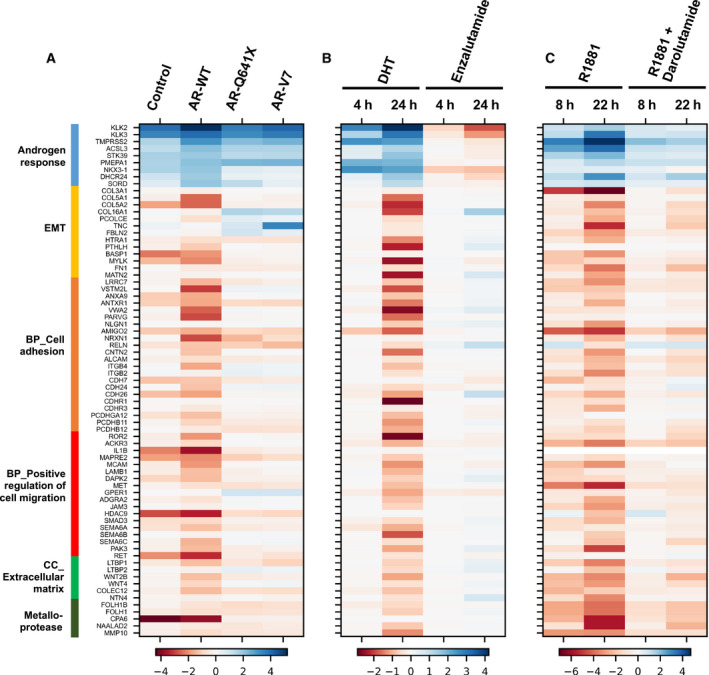
Expression profile of 73 AR‐regulated genes in GSE158557 (A), GSE125014 (B), and GSE148397 (C) data. A selection of 64 genes was used to highlight functional consequences of AR inhibition or the expression of constitutively active AR variants on epithelial‐to‐mesenchymal transition (EMT), cell adhesion and positive regulation of cell migration biological processes (BP), extracellular matrix cellular component (CC), and on metalloproteases among the three RNA‐seq datasets. In addition, nine genes known to be upregulated by AR were used as controls. *DHT, dihydrotestosterone; R1881, synthetic androgen; Enzalutamide and Darolutamide, anti‐androgens*.

These data clearly demonstrated that gene repression by AR could affect significantly cell membranes and cell adhesion features. We further showed that when a panel of 64 AR‐regulated genes involved in these biological processes and molecular functions were chosen, their level of expression was globally higher in the presence of enzalutamide, darolutamide, or in the presence of constitutively active AR variants (Fig. 8), suggesting a similar transcriptional profiling resulting from AR inhibition or the expression of constitutively active AR.

## 4 Discussion

Androgen receptor (AR) is widely described as an androgen‐dependent transcription factor that plays a critical role during the natural history of prostate cancer. AR contributes to the upregulation of key genes for prostate cancer progression [51, 52, 53]. However, few studies have focused on the transcriptional repressive function of AR. In this study, genomic activities of wild‐type AR (AR‐WT) were compared with those of AR‐Q641X and AR‐V7, two constitutively active AR variants that are associated with castration‐resistant prostate cancer. We report here a duality in the repressive function of AR‐WT and constitutively active AR variants. Indeed, compared with DHT‐activated AR‐WT, the number of repressed genes markedly dropped in the presence of AR‐Q641X and AR‐V7, suggesting that transcriptional repressive function by AR‐WT could be pathologically lost in the context of constitutively active AR variants that are devoid of the ligand‐binding domain and AF‐2.

Androgen receptor genomic activity relies on different key steps including androgen binding, nuclear translocation, AR binding as homodimers to AREs localized in enhancer, superenhancer, intron and/or promoter, recruitment of pioneer factors and cofactors for chromatin remodeling, and ultimately transcriptional control of target genes. While the different mechanisms that link AR to the upregulation of target genes have been widely described [7, 8, 9, 52], molecular mechanisms associated with AR‐repressive function are less studied. Also, at the cellular level, functional consequences of AR‐repressive role remain poorly studied.

It has been reported that AR transcriptional repressive function requires DNA binding [54]. However, no consensus negative ARE has been associated with this AR‐repressive function so far [48]. It has been tempting to take advantage of our list of repressed genes in the presence of DHT‐activated AR‐WT to question publicly available AR ChIP‐seq datasets for putative negative ARE. Indeed, the analysis of 500 bp genomic sequences around peak summits from GEO datasets GSE121021 and GSE148358 [31, 55] with MEME‐CHIP program [56] revealed AR and FOXA1 motifs as the two most represented motifs among AR‐binding sites for both the panel of down‐ and upregulated genes. This suggests that, as far as we can conclude from available AR cistrome datasets, gene repression by a full‐length AR does not rely on binding to peculiar AREs. Besides, it has been reported that constitutively active AR variants display their own cistrome [57, 58, 59]. Consequently, it remains to determine whether particular pioneer factor recruitment and distinctive chromatin conformation around downregulated genes could explain the duality between full‐length AR and constitutively active AR for gene repression.

Androgen receptor transcriptional repressive function could also be associated with its ability to recruit repressive complexes causing chromatin inaccessibility [48, 60, 61, 62]. AR transcriptional activities can be negatively controlled by histone deacetylases (HDACs), such as HDAC1, HDAC2, NCoR/SMRT, or SIRT [4, 63, 64]. AR interacts with the histone lysine methylase EZH2 that catalyzes H3K27me3 and H3K4me3 repressive marks [65]. Changes in the level of expression of these key epigenetic markers could potentially be a mechanism associated with the loss of repressive capacity of constitutively active AR variants. Such gene expression deregulation was not evidenced in our data as the level of expression of AR corepressors remained mainly in the gray nonsignificant area in volcano‐plots representing the distribution of RNA‐seq data of our four experimental conditions (Fig. 2). A differential coregulator recruitment could be another mechanism that could be relied to the decreased transcriptional repressive capacities observed with constitutively active AR variants. AR C‐terminal part englobing the LBD and AF‐2 largely contributes to cofactor recruitment. The loss of LBD and AF‐2 in constitutively active AR variants could affect corepressor recruitment. The BioID2 approach by biotinylating proteins that interacted directly or indirectly, or were within proximity (~ 10 nm) to DHT‐activated AR‐WT, AR‐Q641X or to AR‐V7 led us to highlight a lower recruitment of corepressors by constitutively active AR variants. Further technology and cellular models are required to investigate more deeply this property.

At the cellular level, functional consequences of full‐length AR and constitutively active AR variant transcriptional activities have been relatively described [25, 66, 67, 68, 69]. However, cellular consequences of AR‐repressive function in prostate cancer cells remain elusive. Indeed, ontology analysis of genes downregulated in LNCaP following the addition of R1881 for 24 h reveals uniquely ‘*signal transducer activity*’ as the most represented functional category among R1881 downregulated genes [70]. In LNCaP cell line model again, Zhao et al. [65] suggest that AR‐repressed genes are developmental regulators involved in cell differentiation. The functional characterization of the panel of downregulated genes in PC3 prostate cancer cells that do not express AR, following transfection with a full‐length wild‐type AR, includes GO terms involved in transport and cellular localizations, and in general metabolic process such as the tricarboxylic acid cycle, which is according to the authors consistent with a growth inhibition phenotype [71]. In the VCaP prostate cancer cell model, Gao et al. [72] used AR ChIP‐seq and transcriptome profiling to identify genes required for DNA replication as highly enriched among androgen‐repressed genes. So, in brief, few functional analyses on genes repressed by AR have been reported so far. Here, our data indicate that at the cellular level, AR‐WT repressive function could significantly target genes involved in cell adhesion, which was not the case with constitutively active AR variants.

Consistent with previous data, our data also indicate that constitutively active AR variants can upregulate the expression of genes involved in epithelial‐to‐mesenchymal transition (EMT) [25, 26, 68, 73, 74]. EMT is crucial in PCa progression and resistance to castration [75, 76, 77, 78, 79, 80, 81]. In PCa, EMT emerges as a result of selection pressure of full‐length AR inhibition during castration [25, 26, 68, 73, 82, 83, 84, 85].

## 5 Conclusions

Altogether, these observations support a model in which androgens and full‐length AR signaling negatively regulate EMT in epithelial prostate cells. Besides, constitutively active AR variants would pathologically upregulate EMT genes to promote tumor progression. So, we believe that the transcriptional repressive program of the full‐length wild‐type AR in prostate cancer is determinant for epithelial cell behavior and inhibition of tumor progression. Consequently, the systematic targeting of full‐length AR in prostate cancer deserves attention.

## Conflict of interest

The authors declare no conflict of interest.

## Author contributions

JC, EE, and POMB designed the experiment. POMB and EE performed the experiments up to RNA‐seq. EE, POMB, ZA, and BK realized RNA‐seq analysis. EE, JC, BM, and LN realized proteomic analysis. JC, EE, POMB, FC, and ES cowrote the publication.

### Peer Review

The peer review history for this article is available at https://publons.com/publon/10.1002/1878‐0261.13164.

## Supporting information


**Fig. S1**. Validation of experimental conditions. (A) Schematic view of performed experiments. (B) Western blot performed in LNCaP transduced cells showing expression of endogenous AR in Control and co‐expression with EGFP‐tagged AR‐WT (AR‐WT), EGFP‐tagged AR‐Q641X (AR‐Q641X) or EGFP‐tagged AR‐V7 (AR‐V7). (C) Heatmap showing the activation of known AR‐regulated genes upon AR activation in the four experimental conditions. AR, androgen receptor; DHT, dihydrotestosterone; EtOH, ethanol (vehicle).

**Fig. S2**. Analysis of ARv567es transcriptional activity in LNCaP cells. A doxycycline‐inducible expression system (GEO datasets GSE 125014) was used to analyze transcriptomic changes mediated by ARv567es in LNCaP cells. (A) Volcano plot represents the distribution of differential gene expression calculated between doxycycline‐treated cells and vehicle‐treated cells in the absence of androgen. Genes with adjusted *P*‐value < 0.05 and |log_2_FC| > 1 are shown in red (significantly down‐regulated genes) and blue (significantly up‐regulated genes). (B) Gene Set Enrichment Analysis of the LNCaP‐ARv567es expressing cells data showing significant enrichment for “androgen response”, “epithelial mesenchymal transition” and “apical junction” gene sets (NES: Normalized enrichment score). All enrichment scores have a nominal *P*‐value = 0 and an FDR q‐value < 0.005.

**Fig. S3**. Validation of transcriptional repression activity of AR in C4‐2B cells by qPCR. The log2Fold change in gene expression were calculated between the four experimental conditions and the control (eGFP) cells treated with vehicle as reference. Bar graphs represent mean of 3 biological repeats. Student’s t‐test was used to compare control, AR‐WT, AR‐Q641X or AR‐V7 condition with the eGFP condition treated with vehicle. *P < 0.05, **P < 0.01, ***P < 0.001, ****P < 0.0001, ns, non‐significant.

**Fig. S4**. RNA‐seq and mass spectrometry (MS) cross‐analysis of AR‐WT repressive activity. (A) Among the 395 down‐regulated genes, only 14 were identified by MS. (B) Volcano plot representing the distribution of MS data and cross‐analysis with RNA‐seq. Proteins with adjusted *P*‐value < 0.05 and |log_2_FC| > 0.6 are shown in red (significantly under‐represented proteins) and blue (significantly over‐represented proteins). Number of differentially represented proteins are indicated below the plots. The 14 down‐represented proteins at 24 and 48 h after DHT treatment are shown in green.

**Fig. S5**. Pipeline for RNA‐seq/ChIP‐seq intersection and motif analysis. AR ChIP‐seq data available in narrowPeak file format were downloaded from the Gene Expression Omnibus (GEO) database. Sample GSM3424005 [55] referring to AR ChIP‐seq from LNCaP cells cultured in complete medium provides 21127 AR binding sites (peaks). Samples GSM4462682, GSM4462683 and GSM4462684 [31] corresponding to three replicates of VCaP cells treated with 1nM of R1881 for 22h were first subjected to the bedtools intersect function from pybedtools library on python 3 to identify 30941 AR peaks common to the three replicates. Then, the Genomic Regions Enrichment of Annotations Tool (GREAT version 4.0.4) program was used to associate the AR binding sites to putative target genes with the single nearest method and 1000 kb as the maximum extension (http://great.stanford.edu/public/html). Intersection of these ChIP‐seq AR target genes with genes identified as differentially expressed in RNA‐seq data provided us a list of AR peaks associated with genes up‐regulated or down‐regulated by AR in LNCaP and VCaP cells. In order to proceed to motif analysis, sequences corresponding to AR binding sites (500 bp centered on the peak summit) were retrieved from human reference genome (hg19/GRCh37) and submitted to MEME‐ChIP webserver (https://meme‐suite.org/meme/tools/meme‐chip).

**Fig. S6**. Motif analysis of AR peaks in LNCaP and VCaP cells. The first two DNA sequence motifs found by the MEME‐ChIP program in AR binding sites associated to up‐regulated and down‐regulated genes by AR in LNCaP cells (A) and in VCaP cells (B).

**Fig. S7**. BioID cellular model to investigate difference in partner recruitment between DHT‐activated AR‐WT and constitutively active AR variants. (A) Western blot showing expression of myc‐BioID2 (Control) and myc‐BioID2‐AR in the presence of doxycycline (DOX) in stable transduced LNCaP cells. (B and C) Validation of functionality of myc‐BioID2‐AR‐WT construct in transduced LNCaP cells. (B) Streptavidin‐HRP labelling revealed protein biotinylation induced by myc‐BioID2 or myc‐BioID2‐AR‐WT in LNCaP cells in the presence or absence of doxycycline, and in the presence of 10 nM DHT. (C) Luciferase assay using the *PSA61*‐luc construct confirmed androgen‐dependent transcriptional activities of Myc‐BioID2‐AR‐WT fusion protein in LNCaP cells. In brief, 10^4^ LNCaP cells were transfected in triplicates with 230 ng of *PSA61*‐luc (kindly provided by Dr. Trapman, Erasmus University, Rotterdam) and 20 ng of Renilla‐luc (pGL4.70, Promega) plasmids using the JetPEI transfection reagent (Polyplus transfection) and according to the manufacturer’s protocol. After 48 hours, luciferase activities were measured using Dual Dual‐Glo® Luciferase Assay System (Promega) following supplier’s instructions. Bar graph represents mean of 3 biological repeats.

**Table S1**. List of primers used for RT‐qPCR.

**Table S2**. Significantly regulated proteins in MS analysis.

**Table S3**. Gene Set Enrichment Analysis down‐regulated pathways.

## Data Availability

All RNA‐seq data are available at NCBI Gene Expression Omnibus (GEO) under GSE158557 (https://www.ncbi.nlm.nih.gov/geo/). Proteomic data are available at the ProteomeXchange Consortium via the PRIDE database under PXD029454 for BioID2 analysis and PXD029520 for kinetic analysis (http://www.ebi.ac.uk/pride).

## References

[1] Ferlay J , Colombet M , Soerjomataram I , Mathers C , Parkin DM , Piñeros M , et al. Estimating the global cancer incidence and mortality in 2018: GLOBOCAN sources and methods. Int J Cancer. 2019;144:1941–53. 30350310 10.1002/ijc.31937

[2] Brinkmann AO , Blok LJ , de Ruiter PE , Doesburg P , Steketee K , Berrevoets CA , et al. Mechanisms of androgen receptor activation and function. J Steroid Biochem Mol Biol. 1999;69:307–13. 10419007 10.1016/s0960-0760(99)00049-7

[3] Keller ET , Ershler WB , Chang C . The androgen receptor: a mediator of diverse responses. Front Biosci J Virtual Libr. 1996;1:d59–71. 10.2741/a116 9159212

[4] Matsumoto T , Sakari M , Okada M , Yokoyama A , Takahashi S , Kouzmenko A , et al. The androgen receptor in health and disease. Annu Rev Physiol. 2013;75:201–24. 23157556 10.1146/annurev-physiol-030212-183656

[5] Gelmann EP . Molecular biology of the androgen receptor. J Clin Oncol. 2002;20:3001–15. 12089231 10.1200/JCO.2002.10.018

[6] Querol Cano L , Lavery DN , Bevan CL . Mini‐review: foldosome regulation of androgen receptor action in prostate cancer. Mol Cell Endocrinol. 2013;369:52–62. 23395916 10.1016/j.mce.2013.01.023

[7] Itkonen H , Mills IG . Chromatin binding by the androgen receptor in prostate cancer. Mol Cell Endocrinol. 2012;360:44–51. 21989426 10.1016/j.mce.2011.09.037

[8] Heemers HV , Tindall DJ . Androgen receptor (AR) coregulators: a diversity of functions converging on and regulating the AR transcriptional complex. Endocr Rev. 2007;28:778–808. 17940184 10.1210/er.2007-0019

[9] Jia L , Berman BP , Jariwala U , Yan X , Cogan JP , Walkers A , et al. Genomic androgen receptor‐occupied regions with different functions, defined by histone acetylation, coregulators and transcriptional capacity. PLoS One. 2008;3:e3645. 18997859 10.1371/journal.pone.0003645 PMC2577007

[10] Jin H , Zhao JC , Wu L , Kim J , Yu J . Cooperativity and equilibrium with FOXA1 define the androgen receptor transcriptional program. Nat Commun. 2014;5:3972. 24875621 10.1038/ncomms4972 PMC4088269

[11] Shang Y , Myers M , Brown M . Formation of the androgen receptor transcription complex. Mol Cell. 2002;9:601–10. 11931767 10.1016/s1097-2765(02)00471-9

[12] Morova T , McNeill DR , Lallous N , Gönen M , Dalal K , Wilson DM , et al. Androgen receptor‐binding sites are highly mutated in prostate cancer. Nat Commun. 2020;11:1–10. 32047165 10.1038/s41467-020-14644-y PMC7012874

[13] Nash C , Boufaied N , Mills IG , Franco OE , Hayward SW , Thomson AA . Genome‐wide analysis of AR binding and comparison with transcript expression in primary human fetal prostate fibroblasts and cancer associated fibroblasts. Mol Cell Endocrinol. 2018;471:1–14. 28483704 10.1016/j.mce.2017.05.006

[14] Pomerantz MM , Li F , Takeda DY , Lenci R , Chonkar A , Chabot M , et al. The androgen receptor cistrome is extensively reprogrammed in human prostate tumorigenesis. Nat Genet. 2015;47:1346–51. 26457646 10.1038/ng.3419 PMC4707683

[15] Cornford P , Bellmunt J , Bolla M , Briers E , De Santis M , Gross T , et al. EAU‐ESTRO‐SIOG guidelines on prostate cancer. Part II: treatment of relapsing, metastatic, and castration‐resistant prostate cancer. Eur Urol. 2017;71:630–42. 27591931 10.1016/j.eururo.2016.08.002

[16] Evans AJ . Treatment effects in prostate cancer. Mod Pathol. 2018;31:110–21. 10.1038/modpathol.2017.158 29297495

[17] Davies A , Conteduca V , Zoubeidi A , Beltran H . Biological evolution of castration‐resistant prostate cancer. Eur Urol Focus. 2019;5:147–54. 30772358 10.1016/j.euf.2019.01.016

[18] Karantanos T , Evans CP , Tombal B , Thompson TC , Montironi R , Isaacs WB . Understanding the mechanisms of androgen deprivation resistance in prostate cancer at the molecular level. Eur Urol. 2015;67:470–9. 25306226 10.1016/j.eururo.2014.09.049 PMC5301306

[19] Vellky JE , Ricke WA . Development and prevalence of castration‐resistant prostate cancer subtypes. Neoplasia. 2020;22:566–75. 32980775 10.1016/j.neo.2020.09.002 PMC7522286

[20] Céraline J , Cruchant MD , Erdmann E , Erbs P , Kurtz J‐E , Duclos B , et al. Constitutive activation of the androgen receptor by a point mutation in the hinge region: a new mechanism for androgen‐independent growth in prostate cancer. Int J Cancer. 2004;108:152–7. 14618630 10.1002/ijc.11404

[21] Dehm SM , Schmidt LJ , Heemers HV , Vessella RL , Tindall DJ . Splicing of a novel androgen receptor exon generates a constitutively active androgen receptor that mediates prostate cancer therapy resistance. Cancer Res. 2008;68:5469–77. 18593950 10.1158/0008-5472.CAN-08-0594 PMC2663383

[22] Li Y , Yang R , Henzler CM , Ho Y , Passow C , Auch B , et al. Diverse AR gene rearrangements mediate resistance to androgen receptor inhibitors in metastatic prostate cancer. Clin Cancer Res. 2020;26:1965–76. 31932493 10.1158/1078-0432.CCR-19-3023 PMC7165042

[23] Marcias G , Erdmann E , Lapouge G , Siebert C , Barthélémy P , Duclos B , et al. Identification of novel truncated androgen receptor (AR) mutants including unreported pre‐mRNA splicing variants in the 22Rv1 hormone‐refractory prostate cancer (PCa) cell line. Hum Mutat. 2010;31:74–80. 19830810 10.1002/humu.21138

[24] Snow O , Lallous N , Singh K , Lack N , Rennie P , Cherkasov A . Androgen receptor plasticity and its implications for prostate cancer therapy. Cancer Treat Rev. 2019;81:101871. 31698174 10.1016/j.ctrv.2019.05.001

[25] Cottard F , Asmane I , Erdmann E , Bergerat J‐P , Kurtz J‐E , Céraline J . Constitutively active androgen receptor variants upregulate expression of mesenchymal markers in prostate cancer cells. PLoS One. 2013;8:e63466. 23658830 10.1371/journal.pone.0063466 PMC3642121

[26] Cottard F , Madi‐Berthélémy PO , Erdmann E , Schaff‐Wendling F , Keime C , Ye T , et al. Dual effects of constitutively active androgen receptor and full‐length androgen receptor for N‐cadherin regulation in prostate cancer. Oncotarget. 2017;8:72008–20. 29069764 10.18632/oncotarget.18270 PMC5641107

[27] Kim D , Pertea G , Trapnell C , Pimentel H , Kelley R , Salzberg SL . TopHat2: accurate alignment of transcriptomes in the presence of insertions, deletions and gene fusions. Genome Biol. 2013;14:R36. 23618408 10.1186/gb-2013-14-4-r36 PMC4053844

[28] Langmead B , Salzberg SL . Fast gapped‐read alignment with Bowtie 2. Nat Methods. 2012;9:357–9. 22388286 10.1038/nmeth.1923 PMC3322381

[29] Anders S , Pyl PT , Huber W . HTSeq A Python framework to work with high‐throughput sequencing data. Bioinformatics. 2014;31:166–9. 25260700 10.1093/bioinformatics/btu638 PMC4287950

[30] Yuan F , Hankey W , Wu D , Wang H , Somarelli J , Armstrong AJ , et al. Molecular determinants for enzalutamide‐induced transcription in prostate cancer. Nucleic Acids Res. 2019;47:10104–14. 31501863 10.1093/nar/gkz790 PMC6821169

[31] Baumgart SJ , Nevedomskaya E , Lesche R , Newman R , Mumberg D , Haendler B . Darolutamide antagonizes androgen signaling by blocking enhancer and super‐enhancer activation. Mol Oncol. 2020;14:2022–39. 32333502 10.1002/1878-0261.12693 PMC7463324

[32] Nagandla H , Robertson MJ , Putluri V , Putluri N , Coarfa C , Weigel NL . Isoform‐specific activities of androgen receptor and its splice variants in prostate cancer cells. Endocrinology. 2021;162:1–20. 10.1210/endocr/bqaa227 PMC8253248 33300995

[33] Love MI , Huber W , Anders S . Moderated estimation of fold change and dispersion for RNA‐Seq data with DESeq2. Genome Biol. 2014;15:550. 25516281 10.1186/s13059-014-0550-8 PMC4302049

[34] R Core Team . R: a language and environment for statistical computing. Vienna, Austria: R Foundation for Statistical Computing; 2020.

[35] Zhu A , Ibrahim JG , Love MI . Heavy‐Tailed prior distributions for sequence count data: removing the noise and preserving large differences. Bioinformatics. 2019;35:2084–92. 30395178 10.1093/bioinformatics/bty895 PMC6581436

[36] Benjamini Y , Hochberg Y . Controlling the false discovery rate: a practical and powerful approach to multiple testing. J R Stat Soc Series B Stat Methodol. 1995;57:289–300.

[37] Subramanian A , Tamayo P , Mootha VK , Mukherjee S , Ebert BL , Gillette MA , et al. Gene set enrichment analysis: a knowledge‐based approach for interpreting genome‐wide expression profiles. Proc Natl Acad Sci USA. 2005;102:15545–50. 16199517 10.1073/pnas.0506580102 PMC1239896

[38] Liberzon A , Birger C , Thorvaldsdóttir H , Ghandi M , Mesirov JP , Tamayo P . The molecular signatures database hallmark gene set collection. Cell Syst. 2015;1:417–25. 26771021 10.1016/j.cels.2015.12.004 PMC4707969

[39] Ashburner M , Ball CA , Blake JA , Botstein D , Butler H , Cherry JM , et al. Gene Ontology: tool for the unification of biology. Nat Genet. 2000;25:25–9. 10802651 10.1038/75556 PMC3037419

[40] Carbon S , Douglass E , Good BM , Unni DR , Harris NL , Mungall CJ , et al. The Gene Ontology resource: enriching a GOld mine. Nucleic Acids Res. 2021;49:D325–34. 33290552 10.1093/nar/gkaa1113 PMC7779012

[41] Chen EY , Tan CM , Kou Y , Duan Q , Wang Z , Meirelles GV , et al. Enrichr: interactive and collaborative HTML5 gene list enrichment analysis tool. BMC Bioinformatics. 2013;14:128. 23586463 10.1186/1471-2105-14-128 PMC3637064

[42] Kuleshov MV , Jones MR , Rouillard AD , Fernandez NF , Duan Q , Wang Z , et al. Enrichr: a comprehensive gene set enrichment analysis web server 2016 update. Nucleic Acids Res. 2016;44:W90–7. 27141961 10.1093/nar/gkw377 PMC4987924

[43] Kim DI , Jensen SC , Noble KA , Kc B , Roux KH , Motamedchaboki K , et al. An improved smaller biotin ligase for BioID proximity labeling. Mol Biol Cell. 2016;27:1188–96. 26912792 10.1091/mbc.E15-12-0844 PMC4831873

[44] Tyanova S , Temu T , Sinitcyn P , Carlson A , Hein MY , Geiger T , et al. The Perseus computational platform for comprehensive analysis of (prote)omics data. Nat Methods. 2016;13:731–40. 27348712 10.1038/nmeth.3901

[45] Oughtred R , Rust J , Chang C , Breitkreutz B‐J , Stark C , Willems A , et al. The BioGRID database: a comprehensive biomedical resource of curated protein, genetic, and chemical interactions. Protein Sci Publ Protein Soc. 2021;30:187–200. 10.1002/pro.3978 PMC7737760 33070389

[46] Oliphant TE . Python for scientific computing. Comput Sci Eng. 2007;9:10–20.

[47] Nelson PS , Clegg N , Arnold H , Ferguson C , Bonham M , White J , et al. The program of androgen‐responsive genes in neoplastic prostate epithelium. Proc Natl Acad Sci USA. 2002;99:11890–5. 12185249 10.1073/pnas.182376299 PMC129364

[48] Grosse A , Bartsch S , Baniahmad A . Androgen receptor‐mediated gene repression. Mol Cell Endocrinol. 2012;352:46–56. 21784131 10.1016/j.mce.2011.06.032

[49] Balk SP , Knudsen KE . AR, the cell cycle, and prostate cancer. Nucl Recept Signal. 2008;6:e001. 18301781 10.1621/nrs.06001 PMC2254330

[50] Sheng X , Arnoldussen YJ , Storm M , Tesikova M , Nenseth HZ , Zhao S , et al. Divergent androgen regulation of unfolded protein response pathways drives prostate cancer. EMBO Mol Med. 2015;7:788–801. 25864123 10.15252/emmm.201404509 PMC4459818

[51] Mills IG . Maintaining and reprogramming genomic androgen receptor activity in prostate cancer. Nat Rev Cancer. 2014;14:187–98. 24561445 10.1038/nrc3678

[52] Wang F , Koul HK . Androgen receptor (AR) cistrome in prostate differentiation and cancer progression. Am J Clin Exp Urol. 2017;5:18–24. 29181434 PMC5698595

[53] Wang Q , Li W , Liu XS , Carroll JS , Jänne OA , Keeton EK , et al. A hierarchical network of transcription factors governs androgen receptor‐dependent prostate cancer growth. Mol Cell. 2007;27:380–92. 17679089 10.1016/j.molcel.2007.05.041 PMC3947890

[54] He Y , Lu J , Ye Z , Hao S , Wang L , Kohli M , et al. Androgen receptor splice variants bind to constitutively open chromatin and promote abiraterone‐resistant growth of prostate cancer. Nucleic Acids Res. 2018;46:1895–911. 29309643 10.1093/nar/gkx1306 PMC5829742

[55] Ramanand SG , Chen Y , Yuan J , Daescu K , Lambros MB , Houlahan KE , et al. The landscape of RNA polymerase II‐associated chromatin interactions in prostate cancer. J Clin Invest. 2020;130:3987–4005. 32343676 10.1172/JCI134260 PMC7410051

[56] Bailey TL , Boden M , Buske FA , Frith M , Grant CE , Clementi L , et al. MEME Suite: tools for motif discovery and searching. Nucleic Acids Res. 2009;37:W202–8. 19458158 10.1093/nar/gkp335 PMC2703892

[57] Chaytor L , Simcock M , Nakjang S , Heath R , Walker L , Robson C , et al. The pioneering role of GATA2 in androgen receptor variant regulation is controlled by bromodomain and extraterminal proteins in castrate‐resistant prostate cancer. Mol Cancer Res. 2019;17:1264–78. 30833300 10.1158/1541-7786.MCR-18-1231

[58] Chen Z , Wu D , Thomas‐Ahner JM , Lu C , Zhao P , Zhang Q , et al. Diverse AR‐V7 cistromes in castration‐resistant prostate cancer are governed by HoxB13. Proc Natl Acad Sci USA. 2018;115:6810–5. 29844167 10.1073/pnas.1718811115 PMC6042123

[59] Lu J , Lonergan PE , Nacusi LP , Wang L , Schmidt LJ , Sun Z , et al. The cistrome and gene signature of androgen receptor splice variants in castration resistant prostate cancer cells. J Urol. 2015;193:690–8. 25132238 10.1016/j.juro.2014.08.043 PMC4411637

[60] Prescott J , Jariwala U , Jia L , Cogan JP , Barski A , Pregizer S , et al. Androgen receptor‐mediated repression of novel target genes. Prostate. 2007;67:1371–83. 17624924 10.1002/pros.20623

[61] Gritsina G , Gao WQ , Yu J . Transcriptional repression by androgen receptor: roles in castration‐resistant prostate cancer. Asian J Androl. 2019;21:215–23. 30950412 10.4103/aja.aja_19_19 PMC6498738

[62] Cucchiara V , Yang JC , Mirone V , Gao AC , Rosenfeld MG , Evans CP . Epigenomic regulation of androgen receptor signaling: potential role in prostate cancer therapy. Cancers. 2017;9:1–29. 10.3390/cancers9010009 PMC5295780 28275218

[63] Nihan Kilinc A , Sugiyama N , Reddy Kalathur RK , Antoniadis H , Birogul H , Ishay‐Ronen D , et al. Histone deacetylases, Mbd3/NuRD, and Tet2 hydroxylase are crucial regulators of epithelial–mesenchymal plasticity and tumor metastasis. Oncogene. 2019;39:1498–513. 31666683 10.1038/s41388-019-1081-2

[64] Torchy MP , Hamiche A , Klaholz BP . Structure and function insights into the NuRD chromatin remodeling complex. Cell Mol Life Sci. 2015;72:2491–507. 25796366 10.1007/s00018-015-1880-8 PMC11114056

[65] Zhao JC , Yu J , Runkle C , Wu L , Hu M , Wu D , et al. Cooperation between Polycomb and androgen receptor during oncogenic transformation. Genome Res. 2012;22:322–31. 22179855 10.1101/gr.131508.111 PMC3266039

[66] Chang C , Lee SO , Yeh S , Chang TM . Androgen receptor (AR) differential roles in hormone‐related tumors including prostate, bladder, kidney, lung, breast and liver. Oncogene. 2014;33:3225–34. 23873027 10.1038/onc.2013.274

[67] Li Y , Chan SC , Brand LJ , Hwang TH , Silverstein KAT , Dehm SM . Androgen receptor splice variants mediate enzalutamide resistance in castration‐resistant prostate cancer cell lines. Cancer Res. 2013;73:483–9. 23117885 10.1158/0008-5472.CAN-12-3630 PMC3549016

[68] Sun Y , Wang B‐E , Leong KG , Yue P , Li L , Jhunjhunwala S , et al. Androgen deprivation causes epithelial‐mesenchymal transition in the prostate: implications for androgen‐deprivation therapy. Cancer Res. 2012;72:527–36. 22108827 10.1158/0008-5472.CAN-11-3004

[69] Ware KE , Garcia‐Blanco MA , Armstrong AJ , Dehm SM . Biologic and clinical significance of androgen receptor variants in castration resistant prostate cancer. Endocr Relat Cancer. 2014;21:T87–T103. 24859991 10.1530/ERC-13-0470 PMC4277180

[70] Ngan S , Stronach EA , Photiou A , Waxman J , Ali S , Buluwela L . Microarray coupled to quantitative RT–PCR analysis of androgen‐regulated genes in human LNCaP prostate cancer cells. Oncogene. 2009;28:2051–63. 19363526 10.1038/onc.2009.68

[71] Lin B , Wang J , Hong X , Yan X , Hwang D , Cho J‐H , et al. Integrated expression profiling and ChIP‐seq analyses of the growth inhibition response program of the androgen receptor. PLoS One. 2009;4:e6589. 19668381 10.1371/journal.pone.0006589 PMC2720376

[72] Gao S , Gao Y , He HH , Han D , Han W , Avery A , et al. Androgen receptor tumor suppressor function is mediated by recruitment of retinoblastoma protein. Cell Rep. 2016;17:966–76. 27760327 10.1016/j.celrep.2016.09.064 PMC5123835

[73] Kong D , Sethi S , Li Y , Chen W , Sakr WA , Heath E , et al. Androgen receptor splice variants contribute to prostate cancer aggressiveness through induction of EMT and expression of stem cell marker genes. Prostate. 2015;75:161–74. 25307492 10.1002/pros.22901 PMC4270852

[74] Liu G , Sprenger C , Sun S , Epilepsia KS , Haugk K , Zhang X , et al. AR variant ARv567es induces carcinogenesis in a novel transgenic mouse model of prostate cancer. Neoplasia. 2013;15:1009–17. 24027426 10.1593/neo.13784 PMC3769880

[75] Beltran H , Hruszkewycz A , Scher HI , Hildesheim J , Isaacs J , Yu EY , et al. The role of lineage plasticity in prostate cancer therapy resistance. Clin Cancer Res. 2019;25:6916–24. 31363002 10.1158/1078-0432.CCR-19-1423 PMC6891154

[76] Bishop JL , Davies A , Ketola K , Zoubeidi A . Regulation of tumor cell plasticity by the androgen receptor in prostate cancer. Endocr Relat Cancer. 2015;22:R165–82. 25934687 10.1530/ERC-15-0137

[77] Blee AM , Huang H . Lineage plasticity‐mediated therapy resistance in prostate cancer. Asian J Androl. 2019;21:241–8. 29900883 10.4103/aja.aja_41_18 PMC6498731

[78] Culig Z . Epithelial mesenchymal transition and resistance in endocrine‐related cancers. Biochimica et Biophysica Acta‐Mol Cell Res. 2019;1866:1368–75. 10.1016/j.bbamcr.2019.05.003 31108117

[79] Tuerff D , Sissung T , Figg WD . Cellular identity crisis: antiandrogen resistance by lineage plasticity. Cancer Biol Ther. 2017;18:841–2. 28475401 10.1080/15384047.2017.1323599 PMC5710664

[80] Van Leenders GJLH , Schalken JA . Epithelial cell differentiation in the human prostate epithelium: implications for the pathogenesis and therapy of prostate cancer. Crit Rev Oncol Hematol. 2003;46:3–10. 10.1016/s1040-8428(03)00059-3 12850522

[81] Wade CA , Kyprianou N . Profiling prostate cancer therapeutic resistance. Int J Mol Sci. 2018;19:904. 10.3390/ijms19030904 PMC5877765 29562686

[82] Jennbacken K , Tesan T , Wang W , Gustavsson H , Damber JE , Welen K . N‐cadherin increases after androgen deprivation and is associated with metastasis in prostate cancer. Endocr relat cancer. 2010;17:469–79. 20233707 10.1677/ERC-10-0015

[83] Miao L , Yang L , Li R , Nava Rodrigues D , Crespo M , Hsieh J‐T , et al. Disrupting androgen receptor signaling induces Snail‐mediated epithelial‐mesenchymal plasticity in prostate cancer. Cancer Res. 2017;77:3101–12. 28302679 10.1158/0008-5472.CAN-16-2169

[84] Schreyer E , Barthélémy P , Cottard F , Ould Madi‐Berthélémy P , Schaff‐Wendling F , Kurtz J‐E , et al. Androgen receptor variants in prostate cancer. Med Sci (Paris). 2017;33:758–64. 28945566 10.1051/medsci/20173308021

[85] Tanaka H , Kono E , Tran CP , Miyazaki H , Yamashiro J , Shimomura T , et al. Monoclonal antibody targeting of N‐cadherin inhibits prostate cancer growth, metastasis and castration resistance. Nat Med. 2010;16:1414–20. 21057494 10.1038/nm.2236 PMC3088104

