# Supplementary figures and images for: Androgen receptor‐mediated transcriptional repression targets cell plasticity in prostate cancer
Source: Mol Oncol. 2022 Feb 2;16(13):2518–36. doi: 10.1002/1878-0261.13164 (PMC9462842; doi:10.1002/1878-0261.13164)

A

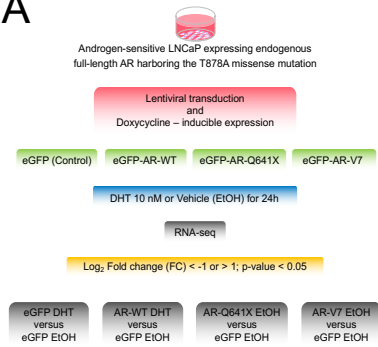

B

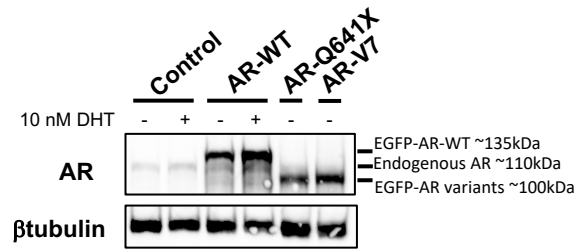

C

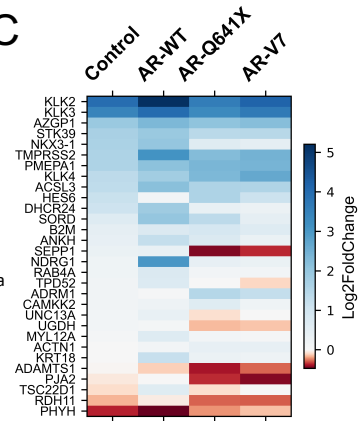

Supplement: Supplementary file 1 — Fig. S1. Validation of experimental conditions. (A) Schematic view of performed experiments. (B) Western blot performed in LNCaP transduced cells showing expression of endogenous AR in Control and co‐expression with EGFP‐tagged AR‐WT (AR‐WT), EGFP‐tagged AR‐Q641X (AR‐Q641X) or EGFP‐tagged AR‐V7 (AR‐V7). (C) Heatmap showing the activation of known AR‐regulated genes upon AR activation in the four experimental conditions. AR, androgen receptor; DHT, dihydrotestosterone; EtOH, ethanol (vehicle). [file MOL2-16-2518-s002.pdf]

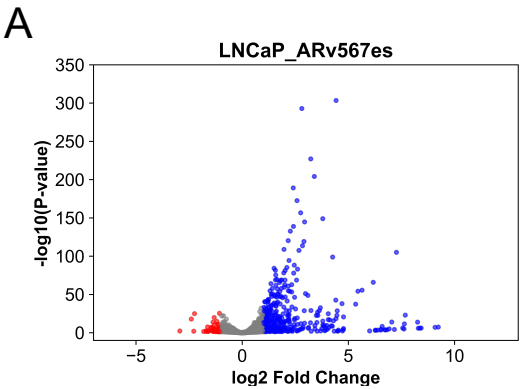

**B**

| <i>HALLMARK</i>                   | <i>NES</i> |
|-----------------------------------|------------|
| Androgen_response                 | 7.57       |
| Epithelial_mesenchymal_transition | 2.38       |
| Apical_junction                   | 2.16       |

Supplement: Supplementary file 2 — Fig. S2. Analysis of ARv567es transcriptional activity in LNCaP cells. A doxycycline‐inducible expression system (GEO datasets GSE 125014) was used to analyze transcriptomic changes mediated by ARv567es in LNCaP cells. (A) Volcano plot represents the distribution of differential gene expression calculated between doxycycline‐treated cells and vehicle‐treated cells in the absence of androgen. Genes with adjusted P‐value < 0.05 and |log2FC| > 1 are shown in red (significantly down‐regulated genes) and blue (significantly up‐regulated genes). (B) Gene Set Enrichment Analysis of the LNCaP‐ARv567es expressing cells data showing significant enrichment for “androgen response”, “epithelial mesenchymal transition” and “apical junction” gene sets (NES: Normalized enrichment score). All enrichment scores have a nominal P‐value = 0 and an FDR q‐value < 0.005. [file MOL2-16-2518-s005.pdf]

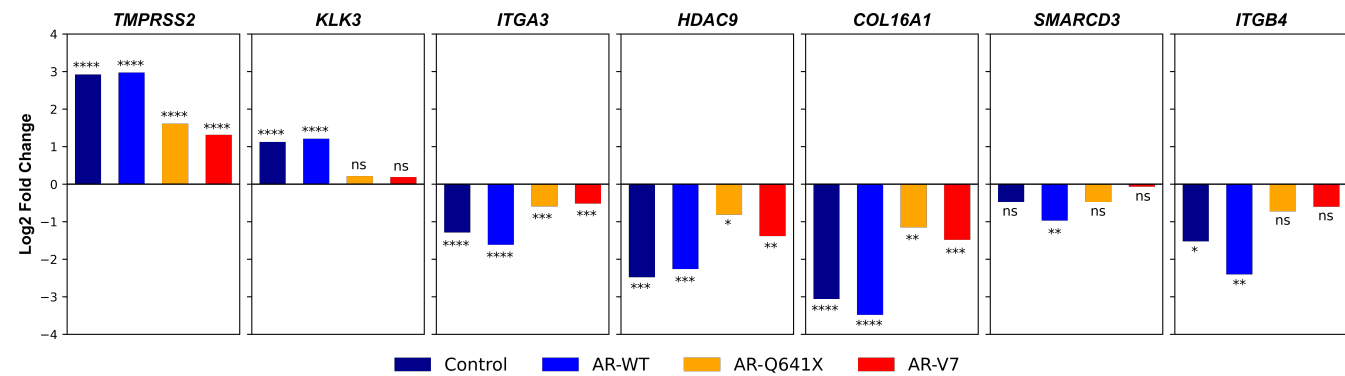

Supplement: Supplementary file 3 — Fig. S3. Validation of transcriptional repression activity of AR in C4‐2B cells by qPCR. The log2Fold change in gene expression were calculated between the four experimental conditions and the control (eGFP) cells treated with vehicle as reference. Bar graphs represent mean of 3 biological repeats. Student’s t‐test was used to compare control, AR‐WT, AR‐Q641X or AR‐V7 condition with the eGFP condition treated with vehicle. *P < 0.05, **P < 0.01, ***P < 0.001, ****P < 0.0001, ns, non‐significant. [file MOL2-16-2518-s001.pdf]

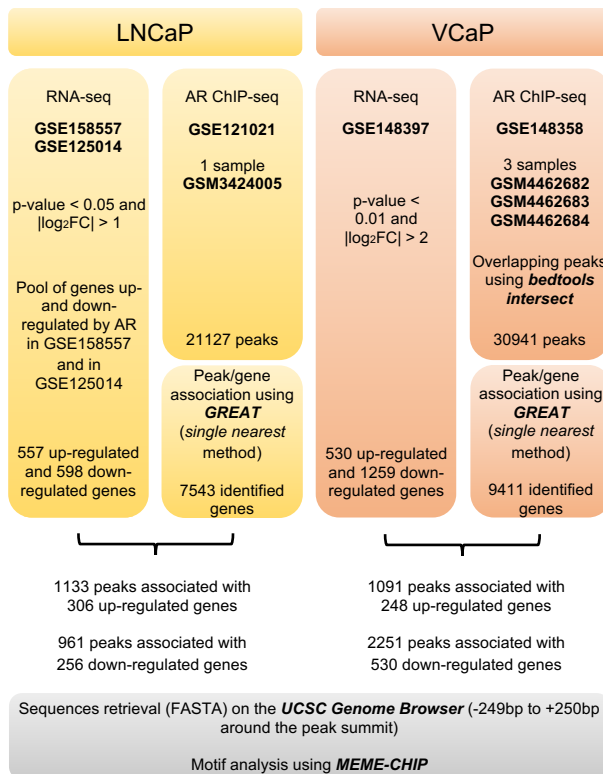

Supplement: Supplementary file 5 — Fig. S5. Pipeline for RNA‐seq/ChIP‐seq intersection and motif analysis. AR ChIP‐seq data available in narrowPeak file format were downloaded from the Gene Expression Omnibus (GEO) database. Sample GSM3424005 [55] referring to AR ChIP‐seq from LNCaP cells cultured in complete medium provides 21127 AR binding sites (peaks). Samples GSM4462682, GSM4462683 and GSM4462684 [31] corresponding to three replicates of VCaP cells treated with 1nM of R1881 for 22h were first subjected to the bedtools intersect function from pybedtools library on python 3 to identify 30941 AR peaks common to the three replicates. Then, the Genomic Regions Enrichment of Annotations Tool (GREAT version 4.0.4) program was used to associate the AR binding sites to putative target genes with the single nearest method and 1000 kb as the maximum extension (http://great.stanford.edu/public/html). Intersection of these ChIP‐seq AR target genes with genes identified as differentially expressed in RNA‐seq data provided us a list of AR peaks associated with genes up‐regulated or down‐regulated by AR in LNCaP and VCaP cells. In order to proceed to motif analysis, sequences corresponding to AR binding sites (500 bp centered on the peak summit) were retrieved from human reference genome (hg19/GRCh37) and submitted to MEME‐ChIP webserver (https://meme‐suite.org/meme/tools/meme‐chip). [file MOL2-16-2518-s009.pdf]

## A LNCaP

## B VCaP

### Up-regulated genes

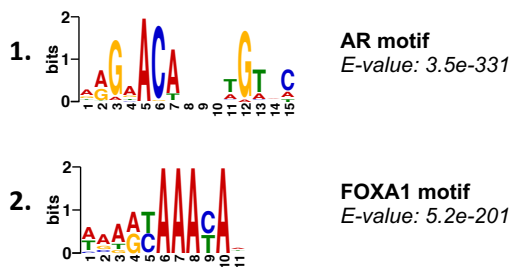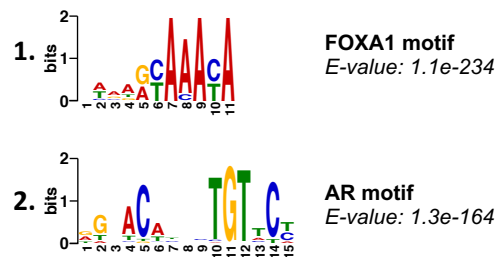

### Down-regulated genes

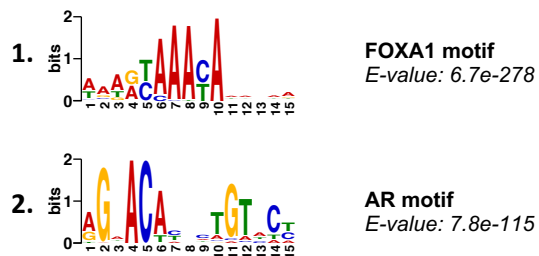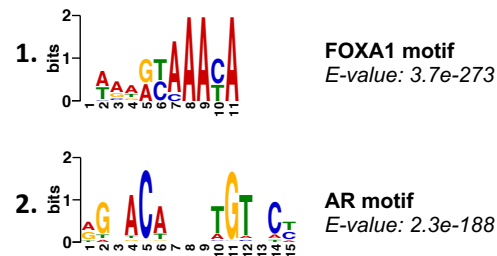

Supplement: Supplementary file 6 — Fig. S6. Motif analysis of AR peaks in LNCaP and VCaP cells. The first two DNA sequence motifs found by the MEME‐ChIP program in AR binding sites associated to up‐regulated and down‐regulated genes by AR in LNCaP cells (A) and in VCaP cells (B). [file MOL2-16-2518-s007.pdf]

A

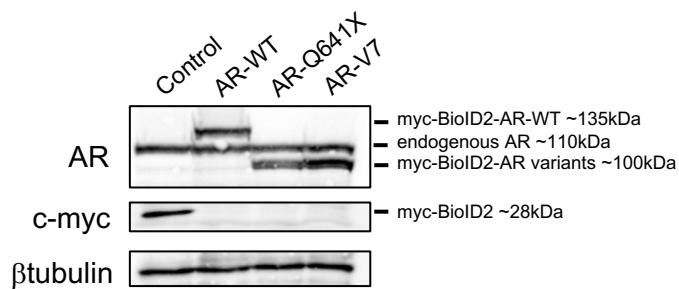

B

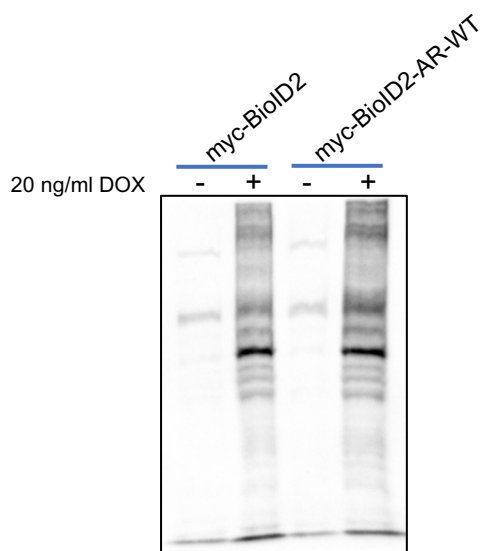

C

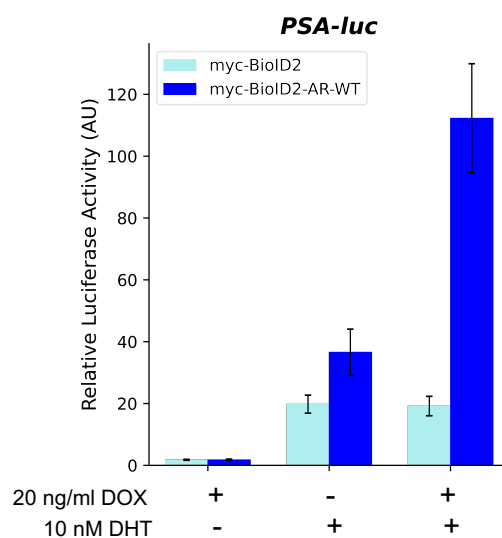

Supplement: Supplementary file 7 — Fig. S7. BioID cellular model to investigate difference in partner recruitment between DHT‐activated AR‐WT and constitutively active AR variants. (A) Western blot showing expression of myc‐BioID2 (Control) and myc‐BioID2‐AR in the presence of doxycycline (DOX) in stable transduced LNCaP cells. (B and C) Validation of functionality of myc‐BioID2‐AR‐WT construct in transduced LNCaP cells. (B) Streptavidin‐HRP labelling revealed protein biotinylation induced by myc‐BioID2 or myc‐BioID2‐AR‐WT in LNCaP cells in the presence or absence of doxycycline, and in the presence of 10 nM DHT. (C) Luciferase assay using the PSA61‐luc construct confirmed androgen‐dependent transcriptional activities of Myc‐BioID2‐AR‐WT fusion protein in LNCaP cells. In brief, 104 LNCaP cells were transfected in triplicates with 230 ng of PSA61‐luc (kindly provided by Dr. Trapman, Erasmus University, Rotterdam) and 20 ng of Renilla‐luc (pGL4.70, Promega) plasmids using the JetPEI transfection reagent (Polyplus transfection) and according to the manufacturer’s protocol. After 48 hours, luciferase activities were measured using Dual Dual‐Glo® Luciferase Assay System (Promega) following supplier’s instructions. Bar graph represents mean of 3 biological repeats. [file MOL2-16-2518-s003.pdf]
