# Supplementary material for: Androgen receptor‐mediated transcriptional repression targets cell plasticity in prostate cancer
Source: Mol Oncol. 2022 Feb 2;16(13):2518–36. doi: 10.1002/1878-0261.13164 (PMC9462842; doi:10.1002/1878-0261.13164)

A

Gene name	Uniprot	24h		48h	
		log2FC	*p*-value	log2FC	*p*-value
TUBB4A	P04350	0.74	2.34E-03	-1.59	1.25E-04
UGT2B15	P54855	-1.28	2.73E-05	-1.02	2.57E-05
MAP1A	P78559	-0.73	1.82E-04	-0.97	1.19E-04
NIPSNAP3A	Q9UFN0	0.42	2.90E-03	-0.93	8.09E-05
TMEM38B	Q9NVV0	-0.20	7.67E-01	-0.91	4.67E-03
MYLK	Q15746	1.31	3.45E-04	-0.74	1.11E-03
GCLC	P48506	-0.23	9.93E-02	-0.67	5.57E-03
UGT2B17	O75795	-0.46	1.70E-05	-0.45	1.39E-04
SELENBP1	Q13228	-0.21	1.26E-02	-0.41	4.20E-05
ATP2B1	P20020	-0.14	2.05E-01	-0.39	5.50E-04
SLC44A1	Q8WWI5	-0.51	9.30E-04	-0.33	1.27E-02
HACL1	Q9UJ83	0.01	8.58E-01	-0.16	3.00E-02
HMGCS2	P54868	-0.84	2.59E-03	-0.04	8.66E-01
PMS2	P54278	0.40	3.17E-01	0.10	2.83E-01

B

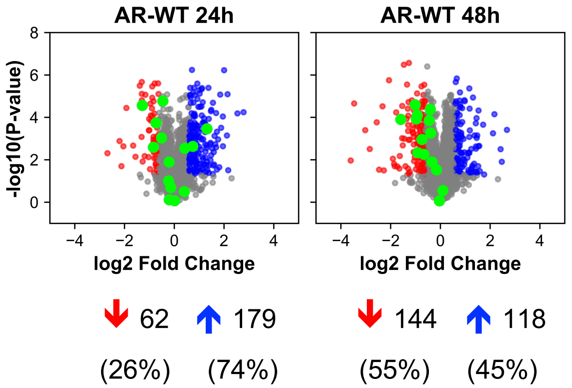

Supplement: Supplementary file 4 — Fig. S4. RNA‐seq and mass spectrometry (MS) cross‐analysis of AR‐WT repressive activity. (A) Among the 395 down‐regulated genes, only 14 were identified by MS. (B) Volcano plot representing the distribution of MS data and cross‐analysis with RNA‐seq. Proteins with adjusted P‐value < 0.05 and |log2FC| > 0.6 are shown in red (significantly under‐represented proteins) and blue (significantly over‐represented proteins). Number of differentially represented proteins are indicated below the plots. The 14 down‐represented proteins at 24 and 48 h after DHT treatment are shown in green. [file MOL2-16-2518-s008.pdf]
