# Supplementary material for: Androgen receptor‐mediated transcriptional repression targets cell plasticity in prostate cancer
Source: Mol Oncol. 2022 Feb 2;16(13):2518–36. doi: 10.1002/1878-0261.13164 (PMC9462842; doi:10.1002/1878-0261.13164)
Supplement: Supplementary file 8 — Table S1. List of primers used for RT‐qPCR. [file MOL2-16-2518-s006.pdf]

Gene name	GenBank accession	GeneGlobe ID	Amplified exons	Amplicon length (pb)
TMPRSS2	NM_001135099	QT00058156	10/11	134
KLK3	NM_001030047	QT00027713	2/3	129
ITGA3	NM_002204	QT00047404	23/24	77
HDAC9	NM_001204144	QT00039333	4/5	92
COL16A1	NM_001856	QT00057435	66/67/68	94
SMARCD3	NM_001003801	QT00069181	12/13	126
ITGB4	NM_000213	QT00049147	2/3/4	102
HMBS	NM_000190	QT00014462	7/8/9	107

Table S1. List of primers used for RT-qPCR
