# Supplementary material for: Androgen receptor‐mediated transcriptional repression targets cell plasticity in prostate cancer
Source: Mol Oncol. 2022 Feb 2;16(13):2518–36. doi: 10.1002/1878-0261.13164 (PMC9462842; doi:10.1002/1878-0261.13164)
Supplement: Supplementary file 9 — Table S2. Significantly regulated proteins in MS analysis. [file MOL2-16-2518-s004.pdf]

Condition	Comparison (A vs B)	Number of differentially represented proteins	Number of under-represented proteins (A<B)	Number of over-represented proteins (A>B)
AR-WT 24h	AR-WT_24hDHT vs AR-WT_24hEtOH	241	62	179
AR-WT 48h	AR-WT_48hDHT vs AR-WT_48hEtOH	262	144	118

Table S2. Significantly regulated proteins in mass spectrometry (MS) analysis. MS results showing number of proteins with adjusted p-value < 0.05 and $|log_2FC| > 0.6$.
