# Supplementary material for: Androgen receptor‐mediated transcriptional repression targets cell plasticity in prostate cancer
Source: Mol Oncol. 2022 Feb 2;16(13):2518–36. doi: 10.1002/1878-0261.13164 (PMC9462842; doi:10.1002/1878-0261.13164)
Supplement: Supplementary file 10 — Table S3. Gene Set Enrichment Analysis down‐regulated pathways. [file MOL2-16-2518-s010.pdf]

Control				AR-WT				AR-Q641X				AR-V7			
HALLMARK	**NES**	**NOM p-val**	**FDR q-val**	***HALLMARK***	**NES**	**NOM p-val**	**FDR q-val**	***HALLMARK***	**NES**	**NOM p-val**	**FDR q-val**	***HALLMARK***	**NES**	**NOM p-val**	**FDR q-val**
HEME_METABOLISM	-2.31	0.00	0.02	*UV_RESPONSE_DN*	-2.29	0.00	0.02	*PROTEIN_SECRETION*	-3.10	0.00	0.00	*PROTEIN_SECRETION*	-2.97	0.00	0.00
COAGULATION	-2.03	0.01	0.05	*KRAS_SIGNALING_DN*	-2.11	0.00	0.03	*MYC_TARGETS_V1*	-2.82	0.00	0.00	*MITOTIC_SPINDLE*	-2.36	0.00	0.00
COMPLEMENT	-1.89	0.01	0.07	*HEME_METABOLISM*	-1.98	0.01	0.05	*E2F_TARGETS*	-2.41	0.00	0.00	*G2M_CHECKPOINT*	-2.12	0.00	0.02
APOPTOSIS	-1.66	0.02	0.17	*COAGULATION*	-1.85	0.01	0.06	*MITOTIC_SPINDLE*	-2.20	0.00	0.01	*HEME_METABOLISM*	-2.11	0.00	0.01
HYPOXIA	-1.62	0.03	0.18	*KRAS_SIGNALING_UP*	-1.69	0.04	0.11	*G2M_CHECKPOINT*	-2.13	0.00	0.01	*UV_RESPONSE_DN*	-2.00	0.01	0.02
INTERFERON_GAMMA_RESPONSE	-1.61	0.04	0.15	*P53_PATHWAY*	-1.69	0.05	0.10	*UV_RESPONSE_DN*	-1.93	0.01	0.02	*E2F_TARGETS*	-1.95	0.00	0.02
APICAL_JUNCTION	**-1.53**	**0.05**	**0.19**	*COMPLEMENT*	-1.61	0.05	0.12	*HEME_METABOLISM*	-1.92	0.00	0.02	*MYC_TARGETS_V1*	-1.83	0.01	0.03
INFLAMMATORY_RESPONSE	-1.47	0.08	0.21	*PANCREAS_BETA_CELLS*	-1.48	0.09	0.18	*SPERMATOGENESIS*	-1.43	0.08	0.17	*SPERMATOGENESIS*	-1.74	0.02	0.05
IL6_JAK_STAT3_SIGNALING	-1.44	0.08	0.21	***APICAL_JUNCTION***	**-1.48**	**0.06**	**0.16**	*KRAS_SIGNALING_UP*	-1.43	0.10	0.15	*KRAS_SIGNALING_UP*	-1.40	0.11	0.21
PANCREAS_BETA_CELLS	-1.38	0.13	0.24	*HEDGEHOG_SIGNALING*	-1.46	0.09	0.16								

Table S3. Gene Set Enrichment Analysis down-regulated pathways. Down-regulated Hallmark gene sets that are significantly enriched (FDR q-val < 0.25) among RNA-seq data for each condition (NES: Normalized enrichment score; NOM p-val: Nominal p-value; FDR q-val: False Discovery Rate q-value).
